# Natural heterogeneous catalysis with immobilised oxidase biocatalysts†

**DOI:** 10.1039/d0ra03618h

**Published:** 2020-05-21

**Authors:** Ashley P. Mattey, Jack J. Sangster, Jeremy I. Ramsden, Christopher Baldwin, William R. Birmingham, Rachel S. Heath, Antonio Angelastro, Nicholas J. Turner, Sebastian C. Cosgrove, Sabine L. Flitsch

**Affiliations:** Department of Chemistry, Manchester Institute of Biotechnology, University of Manchester 131 Princess Street Manchester M1 7DN UK Sebastian.cosgrove@manchester.ac.uk Sabine.flitsch@manchester.ac.uk; Future Biomanufacturing Research Hub, Manchester Institute of Biotechnology, University of Manchester 131 Princess Street Manchester M1 7DN UK

## Abstract

The generation of immobilised oxidase biocatalysts allowing multifunctional oxidation of valuable chemicals using molecular oxygen is described. Engineered galactose oxidase (GOase) variants M_1_ and M_3–5_, an engineered choline oxidase (AcCO6) and monoamine oxidase (MAO-N D9) displayed long-term stability and reusability over several weeks when covalently attached on a solid support, outperforming their free counterparts in terms of stability (more than 20 fold), resistance to heat at 60 °C, and tolerance to neat organic solvents such as hexane and toluene. These robust heterogenous oxidation catalysts can be recovered after each reaction and be reused multiple times for the oxidation of different substrates.

## Abstract 2

The generation of immobilised oxidase biocatalysts allowing multifunctional oxidation of valuable chemicals using molecular oxygen is described.

## Graphical abstract

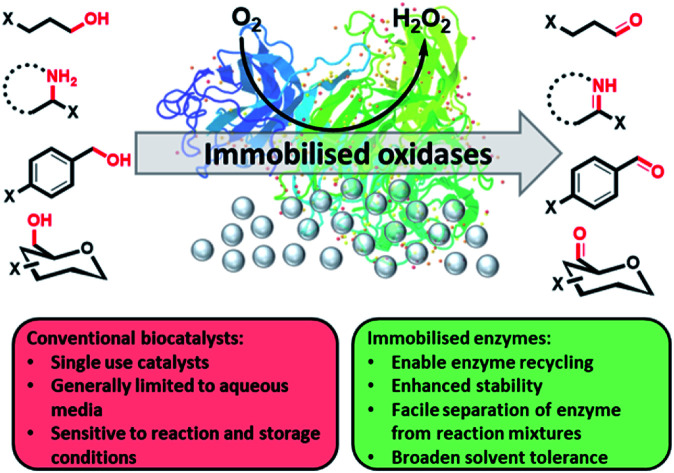

Oxidation comprises one of the most important transformations in bulk synthetic chemistry, with biocatalytic oxidations offering a green alternative to often energy intensive or toxic chemical processes.^1–4^ Although biocatalysis has seen significant developments over the past 20 years, there are still major limitations that prevent widespread use in organic chemistry, mainly owing to their poor stability under process conditions.^5^ Biocatalyst immobilisation offers a base for addressing these limitations, with benefits including increased stability, productivity and solvent tolerance compared with soluble enzymes.^6–9^ Use of immobilised enzymes also allows for ease of scalability and alignment to downstream processes, due to the simplification of catalyst recovery; this includes the potential for continuous biocatalytic flow reactors, with immobilized preparations readily applied in packed bed reactors.^10,11^ Biocatalyst immobilisation offers the selectivity of an enzyme combined with the properties of a carrier, opening the potential for enzymes to be used as reusable heterogeneous catalysts (Fig. 1).^12^ Oxidases, unlike dehydrogenases, are not cofactor dependant and carry out oxidations using molecular oxygen as the sole oxidant with coupled reduction to H_2_O_2_. Particularly attractive oxidase biocatalysts for immobilisation are galactose oxidase (GOase), choline oxidase and monoamine oxidase from *Aspergillus niger* (MAO-N), as all have demonstrated broad applications in biocatalysis.^13–15^ Previous engineering efforts for GOase and choline oxidase have developed a number of variants capable of oxidising a myriad of alcohols and have been demonstrated in both batch and flow reactor configurations,^1,16–20^ including the implementation of GOase in the gram scale biocatalytic manufacture of the investigational anti-HIV drug Islatravir.^21^ Additionally, MAO-N versatility has been extensively shown in amine resolution and functionalization.^15,22^ Here, we demonstrate a general immobilisation strategy that can be applied to a number of oxidase enzymes, enabling multifunctional catalysis that could be readily implemented in industrial processes.

**Fig. 1:**
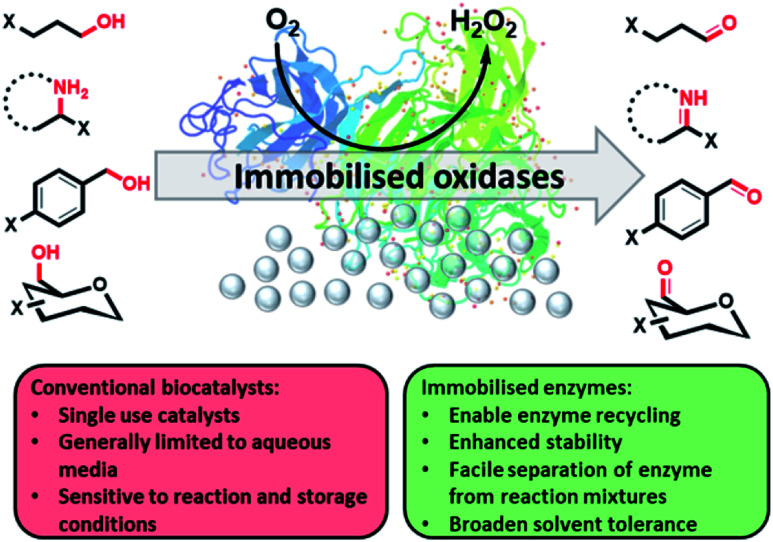
Immobilised oxidases have been used to oxidise a wide range of substrates with an improved tolerance to harsh reaction conditions.

For initial immobilisation studies GOase M_1_ was chosen as the model biocatalyst as it displays potential for large-scale functionalization of complex carbohydrates *via* selective oxidation of the C6–OH of terminal galactose moieties.^23,24^ Pure GOase M_1_ was immobilised on Purolite epoxy/butyl methacrylate beads (ECR8285). Stability of the oxidase was tested throughout the immobilisation process by measuring the activity of the supernatant using the HRP-ABTS assay previously described (ESI†).^16,25^ Protein immobilisation yield was also calculated throughout by spectrophotometric monitoring of the decrease in soluble protein concentration, with a maximum biocatalyst loading of 10 wt% (see ESI† for detailed immobilisation protocol). To test long term stability of the immobilised catalyst we performed multiple biotransformations on the same support over a number of days (Fig. 2). As can be seen below, the half-life (*t*_1/2_) for the soluble enzyme was <24 h, whereas the immobilised preparation still had full activity after 21 days, showing the *t*_1/2_ to be >3 weeks. Furthermore, a sample of the immobilised catalyst was stored at 4 °C for three months and still retained around 50% of the initial activity (see ESI†). It is important to note that the immobilised biocatalyst retained 100% of the activity of its soluble equivalent, and this was the case for the duration of the three week stability experiments (Fig. 2).

**Fig. 2:**
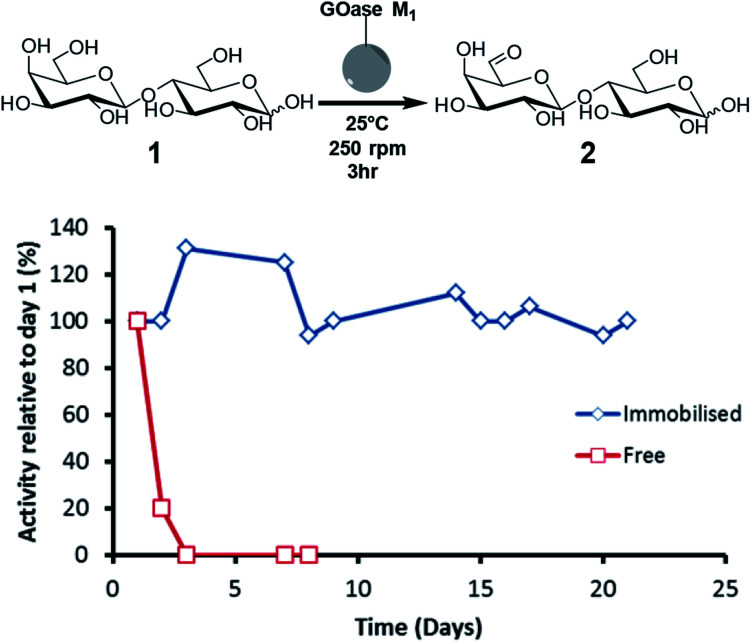
Long term stability of immobilised GOase M_1_ (50 mg, 10 wt%) was tested by running three hour oxidations of 100 mM lactose each day with continuous reuse of the same beads. Catalase and horseradish peroxidase were also added to the reactions.

To further demonstrate this general immobilisation strategy, an engineered choline oxidase (AcCO6),^19^ GOase M_3–5_ and MAO-N D9 were immobilized using the same procedure. Similarly to GOase M_1_, AcCO6, MAO-N D9 and GOase M_3–5_ retained activity for over a week (ESI†). To facilitate oxidation of amines, a previously described blocking strategy was used to prevent nucleophilic amine substrates attaching to the support.^26^

After demonstrating the improved long-term stability and reusability of the immobilised catalysts, the GOase M_1_ preparation was tested for thermal stability and activity (Fig. 3). Reaction temperature has a great impact on the kinetics of immobilised enzymes, with them often being more stable than their soluble counterparts at higher temperatures.^12^ In carbohydrate chemistry, GOase activity at higher temperatures would be a desirable trait in the oxidation and functionalization of polysaccharides due to their poor water solubility at lower temperatures.^27^ Pleasingly, the immobilised GOase outperformed the enzyme in solution, displaying a 50% increase in activity and maintained stability at 60 °C. In comparison, activity of the free enzyme dropped by 50% in addition to a 60% decrease when GOase was preheated at 60 °C and run at room temperature (Fig. 3A).

**Fig. 3:**
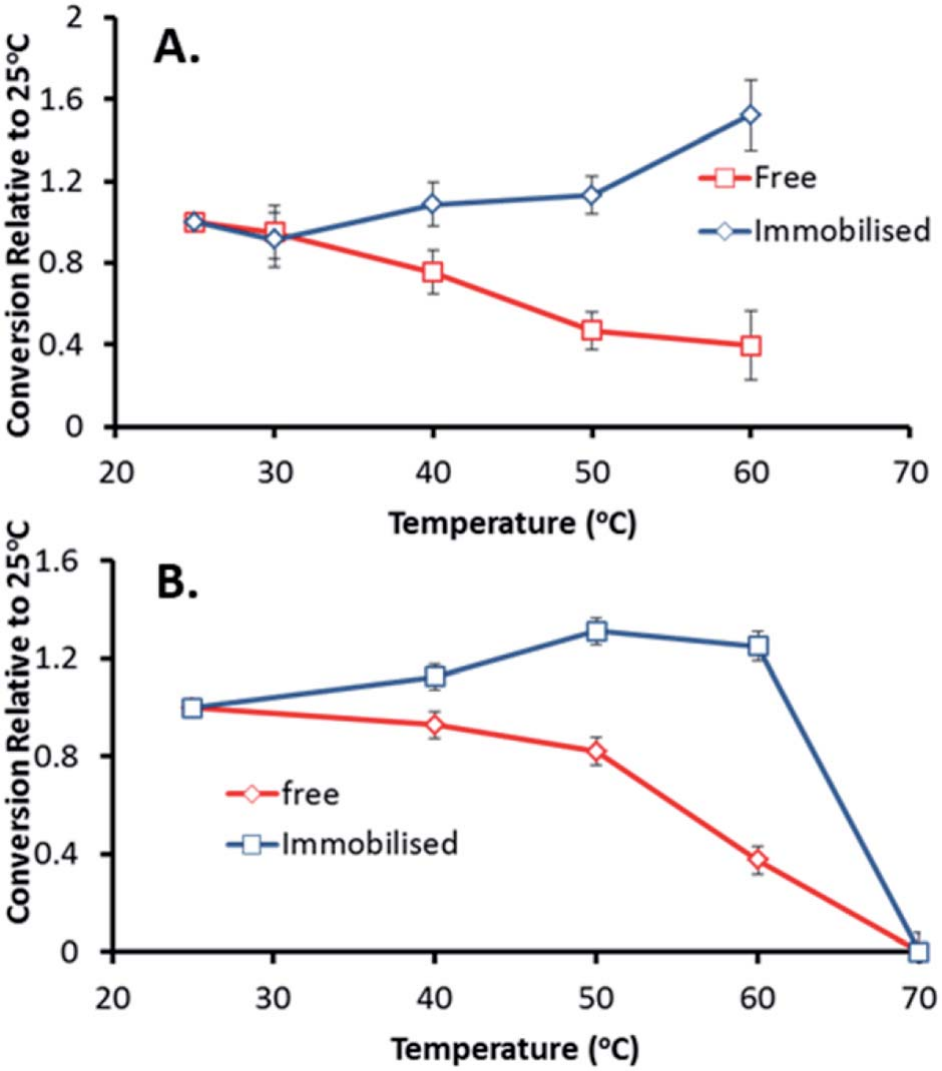
(A) Thermal activity of immobilised GOase M_1_ (100 mg, 1 wt%) and soluble M_1_ (1 mg mL^−1^) was determined through oxidation of 100 mM lactose, conversions were determined by ^1^H NMR analysis and plotted relative to activity at 25 °C. (B) Thermal stability of GOase M_1_ was determined by heating the immobilised catalyst (100 mg, 1 wt%) and soluble enzyme (1 mg mL^−1^) for 17 h. Reaction was then run at 25 °C for 16 h and conversion was determined by ^1^H NMR analysis.

With this increased stability in hand we explored the applicability of the immobilised biocatalysts as reusable, ‘heterogeneous’ catalysts through isolation and reapplication of the same supported biocatalyst preparation for the oxidation of multiple substrates in sequential reactions. Although GOase M_1_ performed well when immobilised, its general use in biocatalysis is limited by its narrow substrate scope (primarily galactosides). Therefore GOase M_3–5_, AcCO6, and MAO-N D9 were selected as they all possess broad substrate scopes.^16,17,19^ Increased use of alcohol oxidases is advantageous as the selective generation of aldehyde intermediates from alcohols has wide applications in the fine chemical industry and as part of biosynthetic cascades.^19,28^ MAOs have been broadly exploited for the resolution and functionalization of amines but can be limited by substrate and product inhibition.^29,30^ Immobilisation of these enzymes could overcome these limitations.^31,32^ Initially GOase M_3–5_ was immobilised at 10 wt% loading and tested against several substituted benzyl alcohols (Table 1). For this procedure the bio-oxidation of benzyl alcohol was carried out and the product 3 removed, the support was then washed with the reaction buffer (with 25% DMSO), then after washing the next substrate was added and the subsequent oxidation was carried out. This process was repeated four times to facilitate oxidation of four different substrates generating 3–6 using the same sample of supported biocatalyst. Immobilised GOase M_3–5_ catalysed extremely efficient oxidations, with >90% conversion achieved within one hour for each substrate (25 mM). Immobilised AcCO6 (10 wt%) was also tested as a recoverable and reusable heterogeneous catalyst, exploiting the broad substrate scope towards aliphatic primary alcohols. Again, the immobilised oxidase was able to achieve good conversions of >78% for all substrates (10 mM) to generate products 7–10 within four hours (Table 1). Finally, MAO-N D9 (10 wt%) was screened against several substrates under the same conditions. Robust biotransformations were demonstrated, fully oxidising achiral substrate 11 and resolving racemic samples 12–14 (20 mM).

**Table 1:** Immobilised oxidases are used to facilitate oxidation of a number of substrates with the same supported catalyst^a^

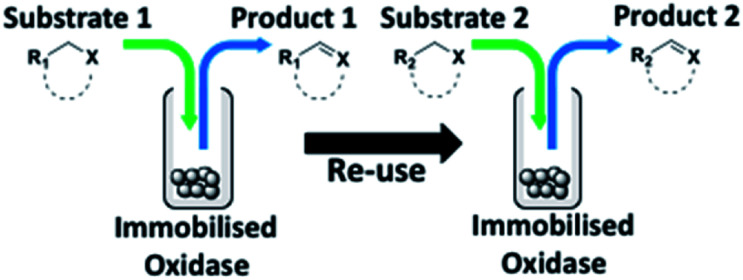			
Day	GOase M_3–5_	AcCO6	MAO-N D9
1	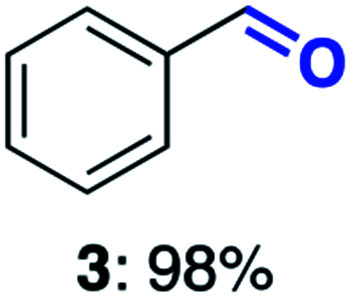	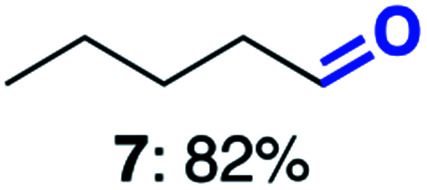	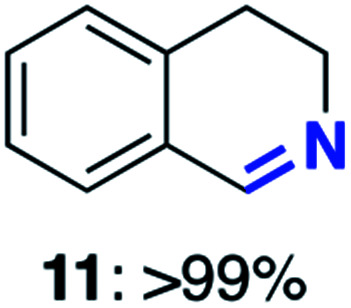
2	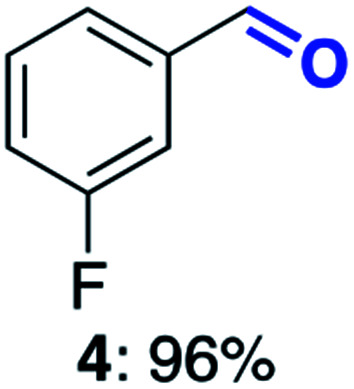	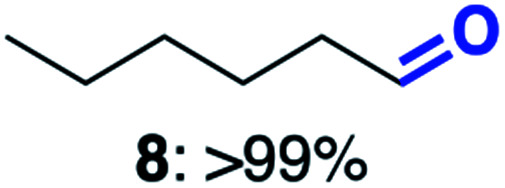	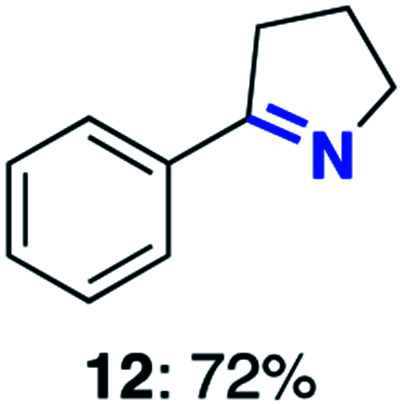
3	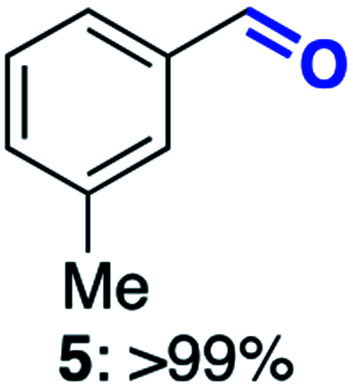	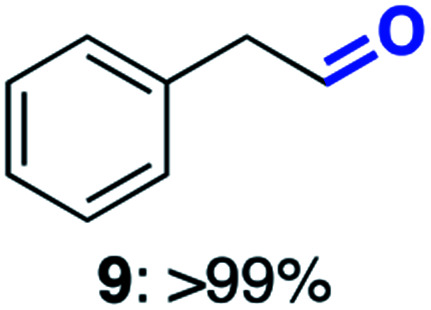	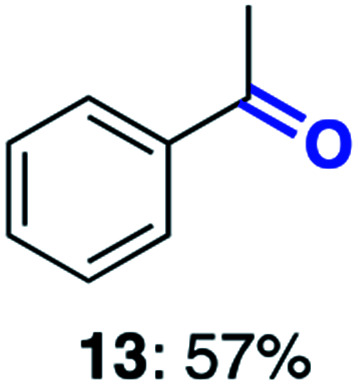
4	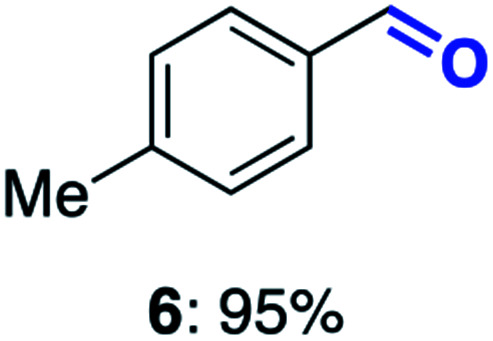	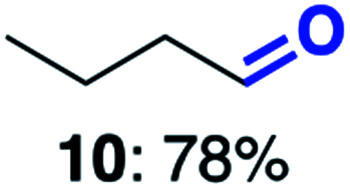	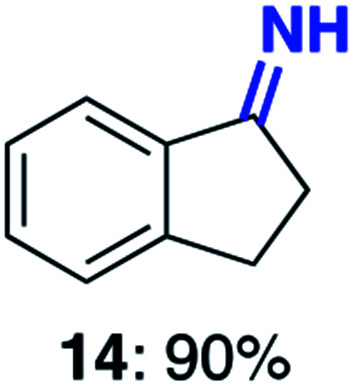

a The same samples of immobilised GOase M_3–5_, AcCO6 and MAO-N D9 were used to sequentially oxidise a number of substrates. Conversions for GOase and AcCO6 were determined by NMR and GC respectively. Conversion for MAO-N were determined for the oxidation of the (*S*)-enantiomer of the racemic substrate (see ESI for reaction conditions).

Next, solvent tolerance was assessed (Fig. 4). Using organic solvents offers a number of advantages over aqueous systems such as increased solubility of hydrophobic substrates and reduction of side product formation.^33^ GOase M_3–5_ demonstrated near identical activity in hexane (aqueous solubility: 14 mg per L H_2_O) and toluene (50 mg per L H_2_O)^34^ to that of the aqueous system in the oxidation of 3-fluorobenzyl alcohol 15. Furthermore, no over oxidation to the corresponding acid was observed.^14^ The use of more water-soluble solvents did have a detrimental effect on activity. AcCO6 performed well in some of the solvents, with a relative activity >40% for EtOAc (8.7 g per L H_2_O), hexane and cyclohexane (5.5 mg per L H_2_O).^34^ MAO-N D9 demonstrated a retained activity of around 30% for all tested solvents. In comparison, the soluble oxidase enzymes displayed no activity when tested in neat solvents.

**Fig. 4:**
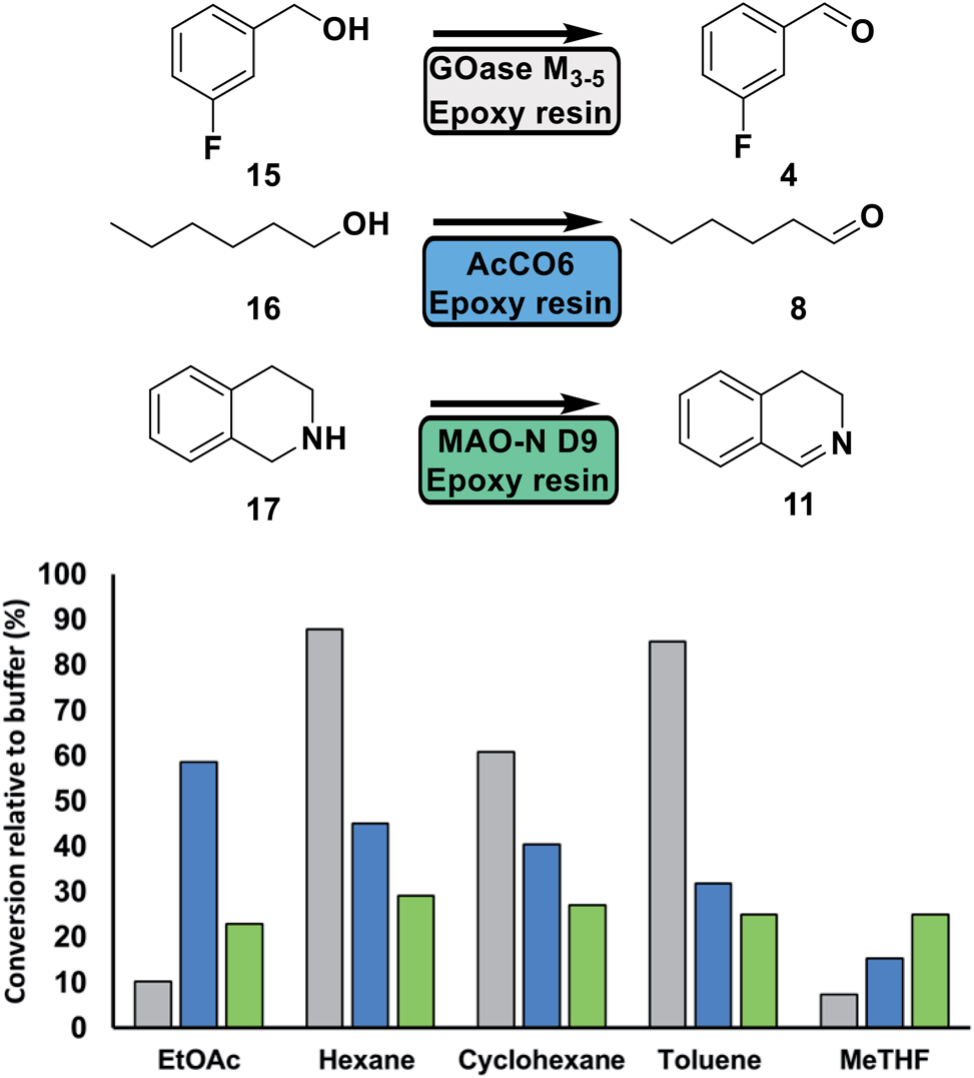
Solvent tolerance of immobilised GOase (Grey), AcCO6 (Blue) and MAO-N D9 (Green) was assessed by drying the immobilised oxidase under reduced pressure, running the biotransformation in neat solvent and plotting relative to buffer (NaPi pH 7.4 GOase, KPi pH 8 AcCO6, KPi pH 8 MAO-N D9). All reactions contained catalase (0.1 mg mL^−1^). Conversions were determined by GC analysis.

There have been several studies of immobilisation of some of the biocatalysts discussed here previously. Rebroš and co-workers used LentiKats polyvinyl alcohol resin to immobilise whole-cell preparations of MAO-N D5.^35^ They demonstrated impressive stabilisation of the immobilised preparations, with samples being stored for 15 months and only showing ∼10% loss in activity. Although immobilisation of whole cells reduces the number of processing steps, ampicillin was required in the storage buffer to prevent bacterial growth, further increasing costs. Additionally, problems may arise with oxygen requirements for cell metabolism limiting the activity of the biocatalyst. The same group showed that crude CFE could also be immobilised using the same support, and demonstrated similarly stability.^36^ We have also demonstrated the immobilisation of AcCO6 previously using controlled porosity glass EziG,^20^ which allows immobilisation through coordination of the histidine tags used for purification of many recombinant biocatalysts.^37^ In the previous study, the use of cyclohexane was also found to be beneficial and viable for AcCO6, with a continuous oxidation of hexanol being run for 20 h with no discernible loss of activity. Whilst no long-term stability studies were undertaken for the affinity resin, the simplicity of the procedure makes this an attractive proposition for immobilisation of His-tagged enzymes. GOase was also immobilised in the multi-enzyme cascade synthesis of islatravir, using affinity resin to improve downstream processing.^21^ Some wild-type GOase and choline oxidase enzymes have been immobilised and applied in biosensors.^38,39^

A summary of the improvements for the immobilised GOase M_1_*versus* the free enzyme is summarised in Table 2.

**Table 2:** Comparison of key properties between immobilised and free GOase M_1_

	Free	Immobilised
Half-life^a^	<24 h	>3 weeks
Thermal activity^b^	0.39	1.52
Thermal stability^c^	0.38	1.25
Solvent tolerance^d^	No	Yes

a Half-life is the point at which the enzyme retains 50% of the original activity.

b Activity measured at 60 °C and is relative to free enzyme activity at 25 °C.

c Stability is the recovered relative activity of the enzyme at 25 °C after incubation at 60 °C for 17 h.

d Solvent tolerance determined as the ability to turn over substrate in neat organic solvent.

In general, biocatalysis adheres to 10 of the 12 principles of green chemistry.^7^ In this study, we show that through immobilisation, the recovery of fully active catalyst is simplified, therefore reducing waste and improving downstream processing in line with the first principle (waste prevention). Furthermore, with efficient catalyst re-use and catalysts stability in storage at room temperature, the energy usage for these immobilised enzymes is significantly reduced, further adhering to the sixth principle of green chemistry (energy efficiency).^7^

In conclusion, we demonstrate the broad application of immobilised oxidases through oxidation of several alcohols and amines with increased solvent tolerance and thermal stability. In particular, the improved stability and reusability make these oxidation catalysts viable options for the organic chemistry community.

## Conflicts of interest

There are no conflicts to declare.

## Supplementary Material

RA-010-D0RA03618H-s001
