# Supplementary material for: Natural heterogeneous catalysis with immobilised oxidase biocatalysts
Source: RSC Adv. 2020 May 21;10(33):19501–5. doi: 10.1039/d0ra03618h (PMC9054114; doi:10.1039/d0ra03618h)
Supplement: RA-010-D0RA03618H-s001 [file RA-010-D0RA03618H-s001.pdf]

Electronic Supplementary Material (ESI) for RSC Advances.
This journal is © The Royal Society of Chemistry 2020

Supporting information

Natural Heterogeneous Catalysis with Immobilised Oxidase Biocatalysts

Ashley P. Mattey,[a] Jack J. Sangster,[a] Jeremy I. Ramsden,[a] Christopher Baldwin,[a] William R. Birmingham,[a] Rachel S. Heath,[a] Antonio Angelastro,[a] Nicholas J. Turner,[a,b] Sebastian C. Cosgrove,*[a,b] Sabine L. Flitsch*[a]

a. Manchester Institute of Biotechnology (MIB) & School of Chemistry, The University of Manchester, 131 Princess Street, Manchester, M1 7DN, United Kingdom.
b. Future Biomanufacturing Research Hub, Manchester Institute of Biotechnology, University of Manchester, 131 Princess Street, Manchester, M1 7DN, UK

Email: *Sebastian C. Cosgrove sebastian.cosgrove@manchester.ac.uk

*Sabine L. Flitsch sabine.flitsch@manchester.ac.uk

Experimental

Materials

HRP (IV) and catalase were purchased from Sigma Aldrich. All reactants and reagents listed were purchased from Sigma Aldrich unless stated otherwise. Lifetech epoxy/butymethacrylate beads (ECR 8285) were purchased from Purolite.

Biocatalyst production

Galactose Oxidase M_1 and $M_{3\text{-}5}$ were expressed and purified as previously described.[1]

Choline Oxidase $AcCO_6$ was expressed and purified as previously described.[2]

MAO-N D9 was expressed and purified as previously described.[3]

Immobilisation procedure for oxidases

Immobilisation buffer: 100 mM sodium phosphate, 300 mM NaCl, pH = 8.0

Immobilisation of oxidases were achieved by following the manufacturers' guidelines available at:

https://www.purolite.com/dam/jcr:3c5b978b-3829-4f92-8654-390863042f09/Lifetech%20ECR%20immobilization%20procedures.pdf

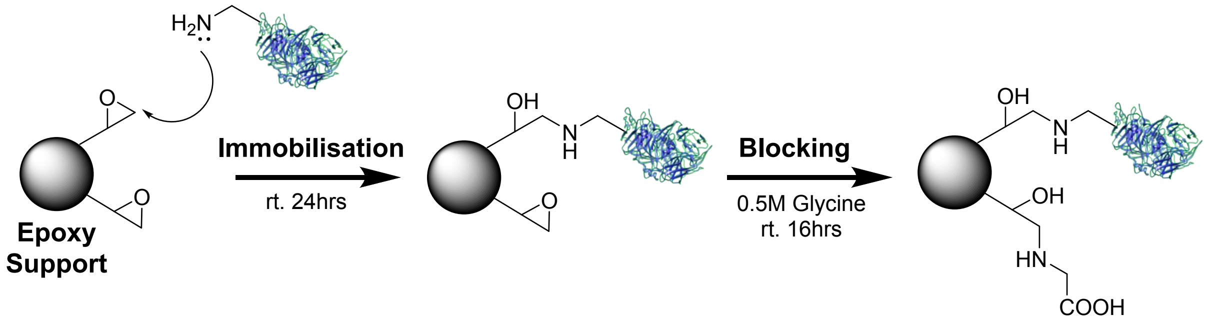

Figure S1: General immobilisation scheme and blocking strategy for epoxy support.

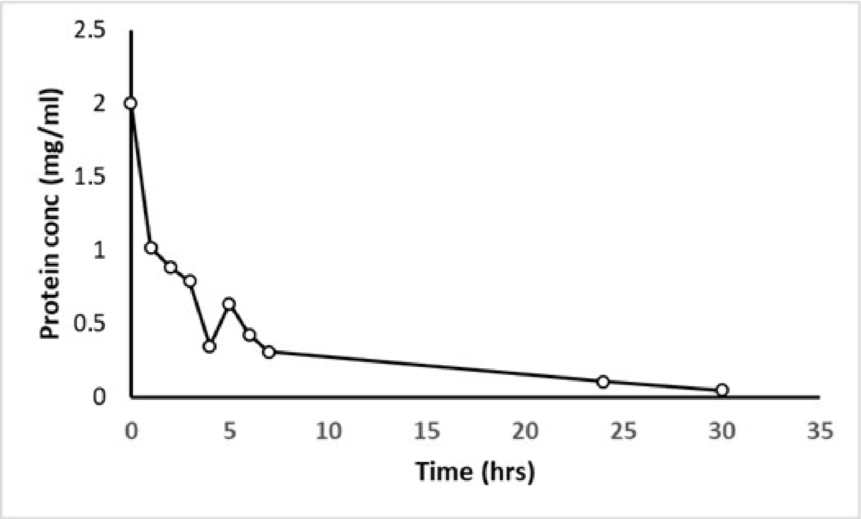

Figure S2: Immobilisation of GOase M_1 onto epoxy methacrylate beads was followed by measuring protein concentration of supernatant throughout immobilisation

Protein Immobilisation yield calculations

Immobilisation Yield:

Initial concentration of enzyme in solution: 3.90 mg mL^{-1}

Final concentration of enzyme in solution: 0.81 mg mL^{-1}

$$\%\,Immobilisation\,Yield = 100 - \left(\frac{Initial\,Enzyme\,Concentration\,in\,Solution}{Final\,Enzyme\,Concentration\,in\,Solution} \times 100\right)$$

$$= 100 - \left(\frac{0.81\,mg\,mL^{-1}}{3.90\,mg\,mL^{-1}} \times 100\right)$$

$$= 79\%$$

Enzyme concentration of solution measured using nanodrop spectrophotometer.

HRP-ABTS assay

Specific activity of GOase M_1 was measured throughout the immobilisation procedure using the ABTS-HRP assay as described.[4] 10ul of the immobilisation supernatant was diluted into NaPi buffer (100 mM, pH 7.4) which contained HRP (0.23 mg mL^{-1}) and ABTS (0.4 mg mL^{-1}) to a final volume of 100 µL. The reaction was initiated with the addition of a stock solution of lactose in water (final substrate concentration 25 mM). Production of the reduced ABTS was measured using a Tecan infinite 200 plate reader at 420 nm and 30 °C for 10 mins. Measurements were made in triplicate.

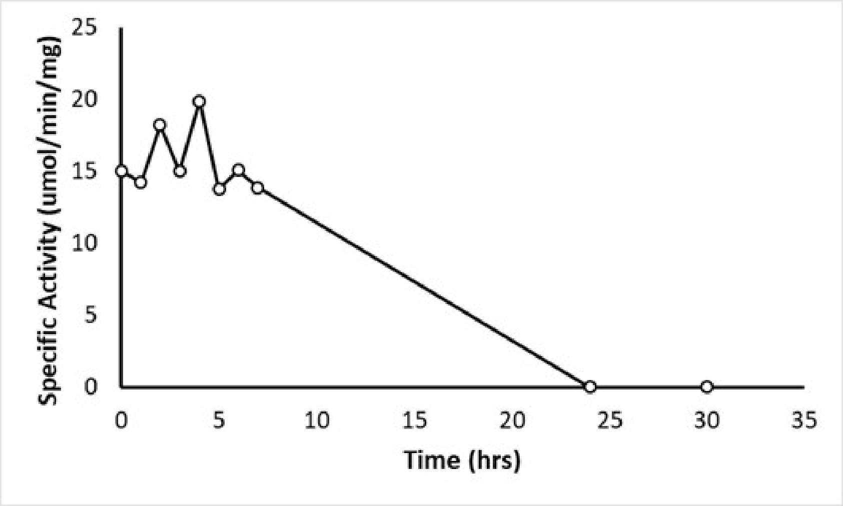

Figure S3: Activity of immobilisation supernatant (GOase M_1 epoxy methacrylate support) was followed using the HRP ABTS assay.

Biotransformation protocols

Long term stability of immobilised GOase M_1

A solution of lactose (100 mM) in NaPi buffer (0.5 mL, 100 mM, pH 7.4) was added to immobilised GOase M_1 (50 mg, 10 wt% GOase), which also contained HRP (0.1 mg mL^{-1}) and Catalase (0.1 mg mL^{-1}). The reaction was carried out in a 2 mL Eppendorf with 250 rpm shaking at 25 °C. After 3 h the supernatant was removed and freeze dried for ^{1}H NMR analysis. The remaining beads were washed with NaPi buffer (0.5 mL $\times$ 4) then stored at 4 °C until the next reaction. This was repeated for each reaction.

General Biotransformations with immobilised GOase M_{3-5}

To a suspension of immobilised GOase M_{3-5} (20 mg, 10 wt%) in NaPi buffer (0.75 mL, 100 mM, pH 7.4) containing HRP (0.1 mg mL^{-1}) and Catalase (0.1 mg mL^{-1}) was added a solution of 3-Me-benzyl alcohol (0.25 mL, 200 mM) in DMSO. The biotransformation was left for 1 h in a 2 mL Eppendorf with 250 rpm shaking at 25 °C, after which, 0.5 mL of supernatant was removed and mixed with 0.1 mL D_2O. ^{1}H NMR spectra were obtained using 400 MHz Bruker instrument with water suppression mode. Conversion was determined by integration of the aldehyde and starting material signals.

General Biotransformations with immobilised AcCO6

To a suspension of immobilised AcCO6 (20 mg, 10 wt%) in KPi buffer (0.375 mL, 100 mM, pH 8) was added a solution of 1-hexanol (0.125 mL, 100 mM) in DMSO. The biotransformation was left for 4 h in a 2 mL Eppendorf with 200 rpm shaking at 30 °C. The supernatant was removed and extracted into MTBE (0.5 mL). Conversion was determined by GC-FID analysis.

General Biotransformations with immobilised MAO-N D9

To a suspension of immobilised MAO-N D9 (20 mg, 10 wt%) in KPi buffer (0.45 mL, 100 mM, pH 8) was added a solution of tetrahydroisoquinoline (0.05 mL, 200 mM) in DMSO. The biotransformation was left for 1 h in a 2 mL Eppendorf with 200 rpm shaking at 30 °C. The supernatant was removed, the pH changed to >12 by addition of NaOH (20 μL, 10 M) and extracted into MTBE (0.5 mL). Conversion was determined by GC-FID analysis.

Thermal stability and activity of GOase M_1

Thermal stability of immobilised GOase M_1 (100 mg, 1 wt%) was determined by heating at a given temperature (30 °C, 40 °C, 50 °C, 60 °C, 70 °C) for 17 h followed by an overnight biotransformation with a solution of lactose (100 mM), HRP (0.1 mg mL^{-1}) and Catalase (0.1 mg mL^{-1}) in NaPi buffer (1 mL, 100 mM, pH 7.4). Conversion was determined by ^{1}H NMR analysis and plotted relative to 25 °C. Thermal activity was determined by running the biotransformation overnight at 30 °C, 40 °C, 50 °C, 60 °C and 70 °C with 250 rpm shaking in a solution of lactose (100 mM), HRP (0.1 mg mL^{-1}) and Catalase (0.1 mg mL^{-1}) in NaPi buffer (1 mL, 100 mM, pH 7.4). Conversion was determined by NMR and plotted relative to 25 °C.

Heterogeneous catalysis with immobilised oxidases

The immobilised oxidase (20 mg, 10 wt%) was prepared and stored in their optimum buffer (pH 7.4 100mM NaPi for GOase M_{3-5} and pH 8 100mM Kpi for AcCO6 and MAO-N D9). Four substrates for each oxidase were prepared in neat DMSO. Each substrate (25 mM for GOase M_{3-5}, 10 mM for AcCO6 and 20 mM MAO-N D9) was incubated with the respective oxidase and after: 1 h for GOase, 4 h for AcCO6 and 16 h for MAO-N D9. The supernatant was removed and either extracted into EtOAc (for GC analysis) or run directly for ^{1}H NMR by mixing with D_2O (GOase reactions). After each substrate the immobilised oxidases were washed with

25% DMSO. The next day the next substrate was then added and the biotransformation analysed.

Oxidase solvent screen

A 20mg (10wt%) sample of immobilised oxidase was dried using centrifugal evaporation to ensure removal of H_2O. After drying, the oxidase was suspended in neat solvent along with the associated substrate. GOase M_{3-5} was incubated with 3-F-benzyl alcohol (0.5 mL, 25 mM) with 250 rpm shaking at 25 °C for 6 h. AcCO6 was incubated with 1-hexanol (0.5 mL, 25 mM) with 200 rpm shaking at 30 °C for 4 h. MAO-N D9 was incubated with tethrahydroisoquinoline (0.5 mL, 20 mM) with 200 rpm shaking at 30 °C for 2 h. The reactions in neat solvent were then directly analysed *via* GC-FID. For reactions in buffer, supernatant was removed and extracted into EtOAc (GOase) or MTBE (AcCO6 and MAO-N D9) and analysed *via* GC-FID.

Analytical methods

GC analysis

GOase and AcCO6: HP-1 (agilent) column (30 m × 0.32 mm × 0.25 μm Method: 40°C to 325°C at 10 °C· min-1 , injector 250 °C, detector 250 °C)

MAO: HP-1 (agilent) column (30 m × 0.32 mm × 0.25 μm Method: 40°C hold for 4 min then 40°C to 200°C at 30 °C· min-1 , injector 250 °C, detector 250 °C)

NMR analysis

NMR experiments were carried out of products. For analysis of carbohydrates the reaction mixtures were concentrated under reduced pressure then re-dissolved in D_2O (600 μL). Spectra were recorded at 400 MHz and 20 °C. For analysis of substituted benzyl alcohols, a 500 μL aliquot of the reaction mixture was removed and diluted with 100 μL D_2O. Spectra were recorded using a water suppression system at 400 MHz and 20 °C.

Spectra

NMR spectra

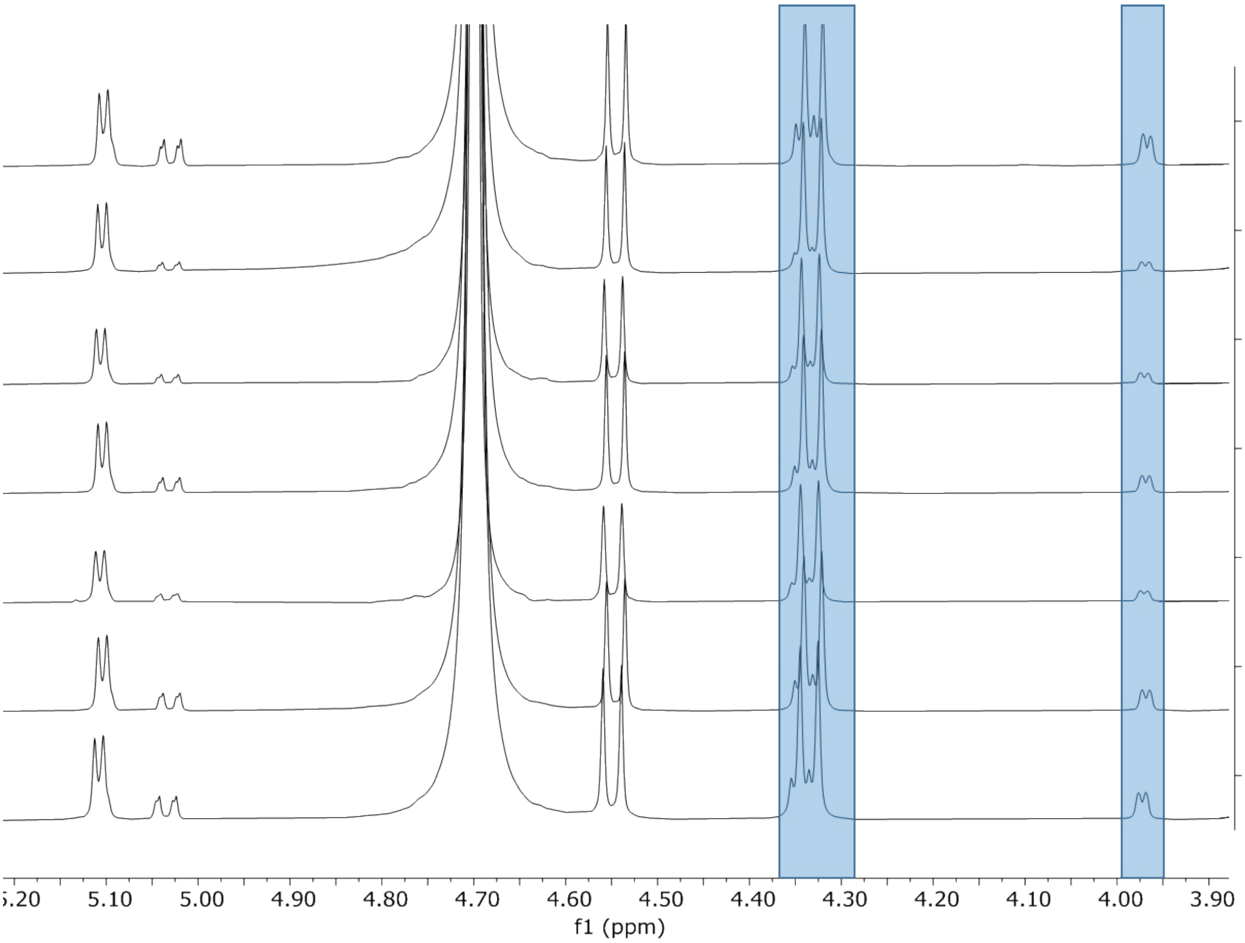

Day	C1 integ	C4 integ	Conversion (%)
1	1	0.16	16
3	1	0.21	21
5	1	0.14	14
7	1	0.14	14
9	1	0.12	12
11	1	0.14	14
15	1	0.16	16

Figure S4: Long term stability of immobilised GOase M_1 (50 mg, 10 wt%) in the 3 h bio-oxidation of 100 mM lactose.

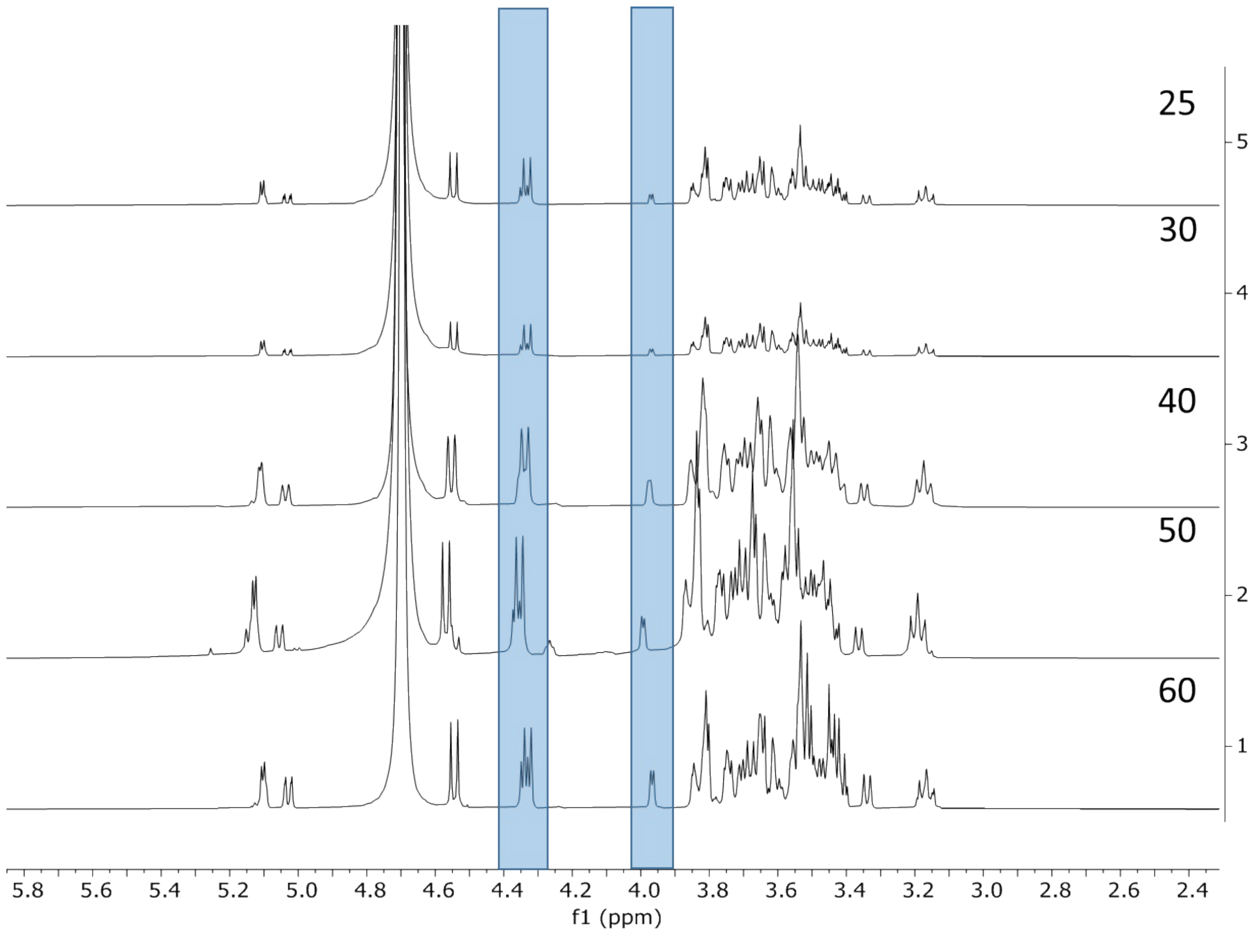

Temp	C1 integ	C4 integ	Conversion (%)
25	1	23	23
30	1	21	21
40	1	25	25
50	1	30	30
60	1	35	35

Figure S5: Thermal activity of immobilised GOase M_1 (100 mg, 1 wt%) in the overnight bio-oxidation of 100 mM lactose.

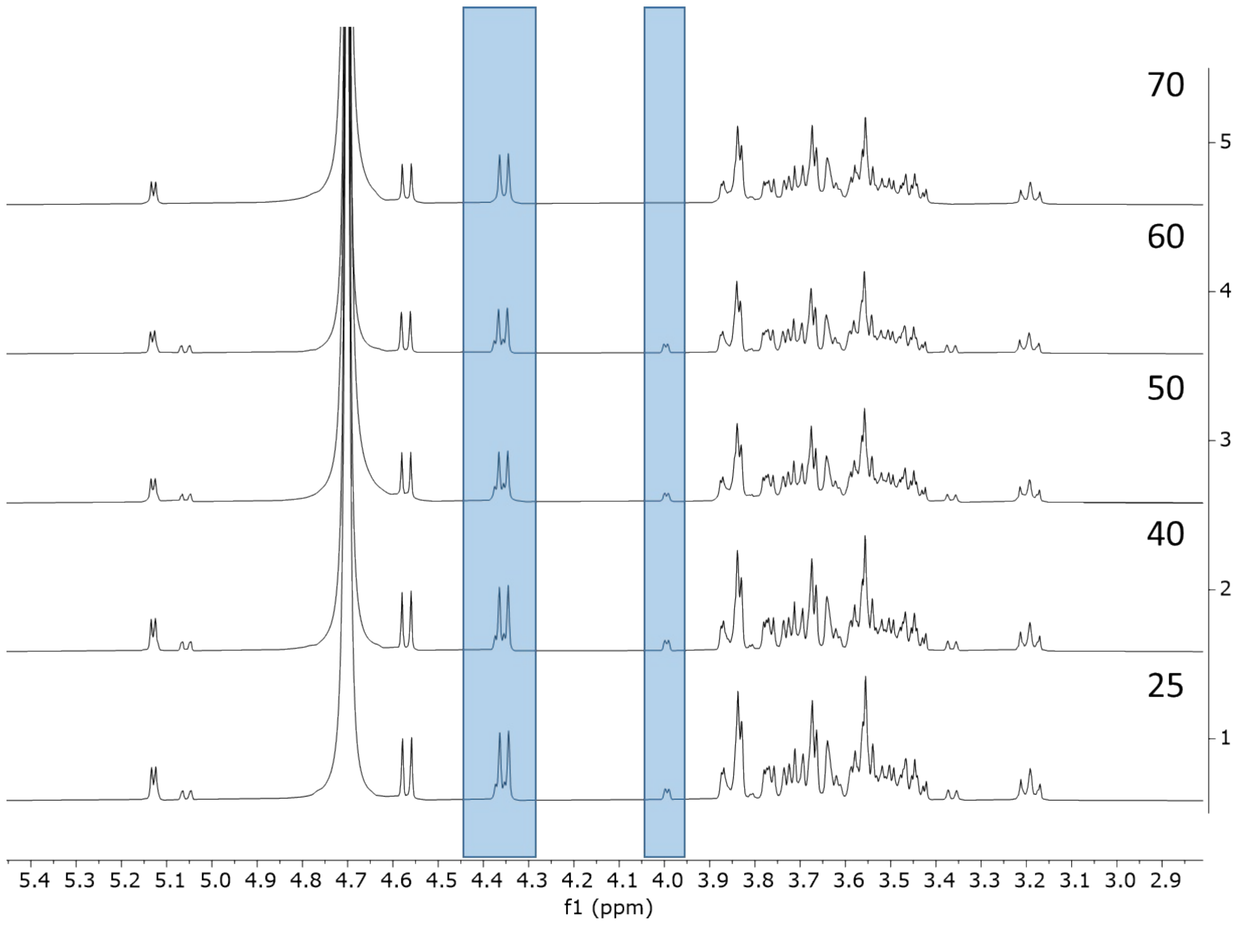

Temp	C1 integ	C4 integ	Conversion (%)
25	1	0.16	16
40	1	0.18	18
50	1	0.20	20
60	1	0.20	20
70	1	0	0

Figure S6: Thermal stability of immobilised GOase M_1 (100 mg, 1 wt%) in the bio-oxidation of 100 mM lactose.

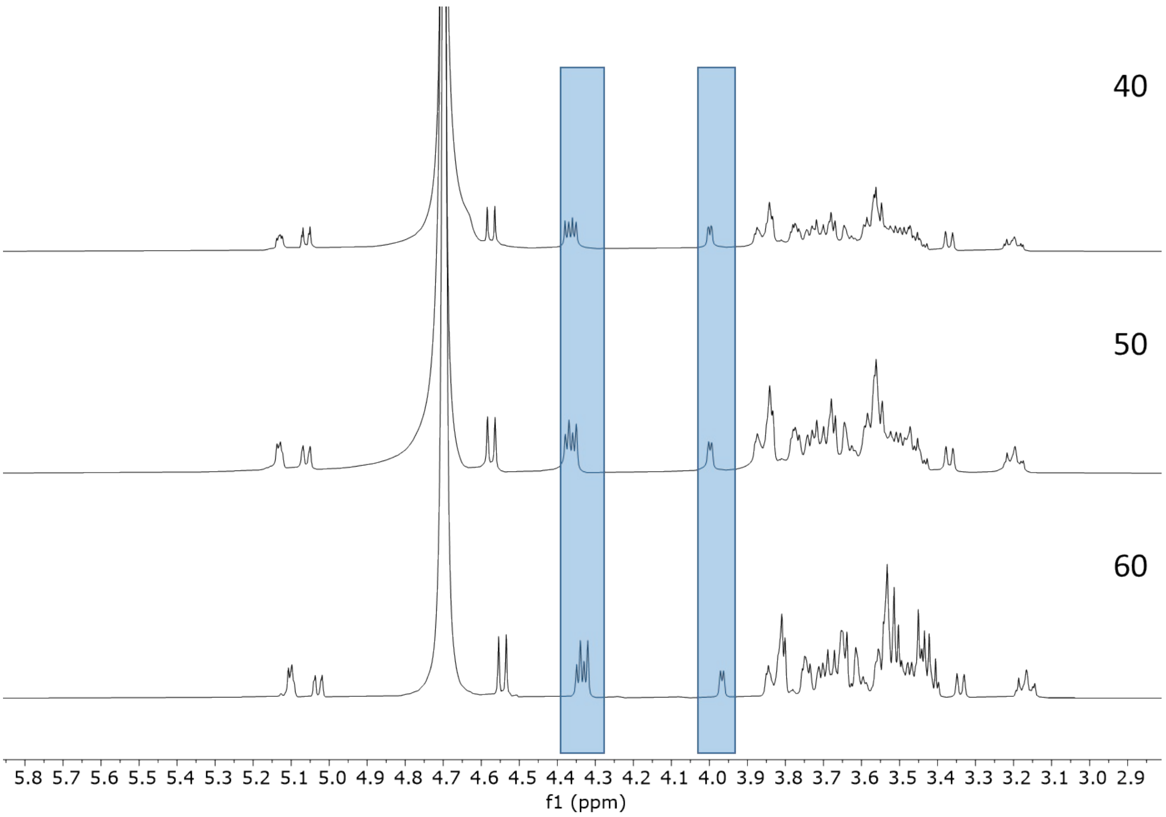

Temp	C1 integ	C4 integ	Conversion (%)
40	1	0.61	61
50	1	0.38	38
60	1	0.32	32

Figure S7: Thermal activity of free GOase M_1 (1 mg mL^{-1}) in the bio-oxidation of 100 mM lactose.

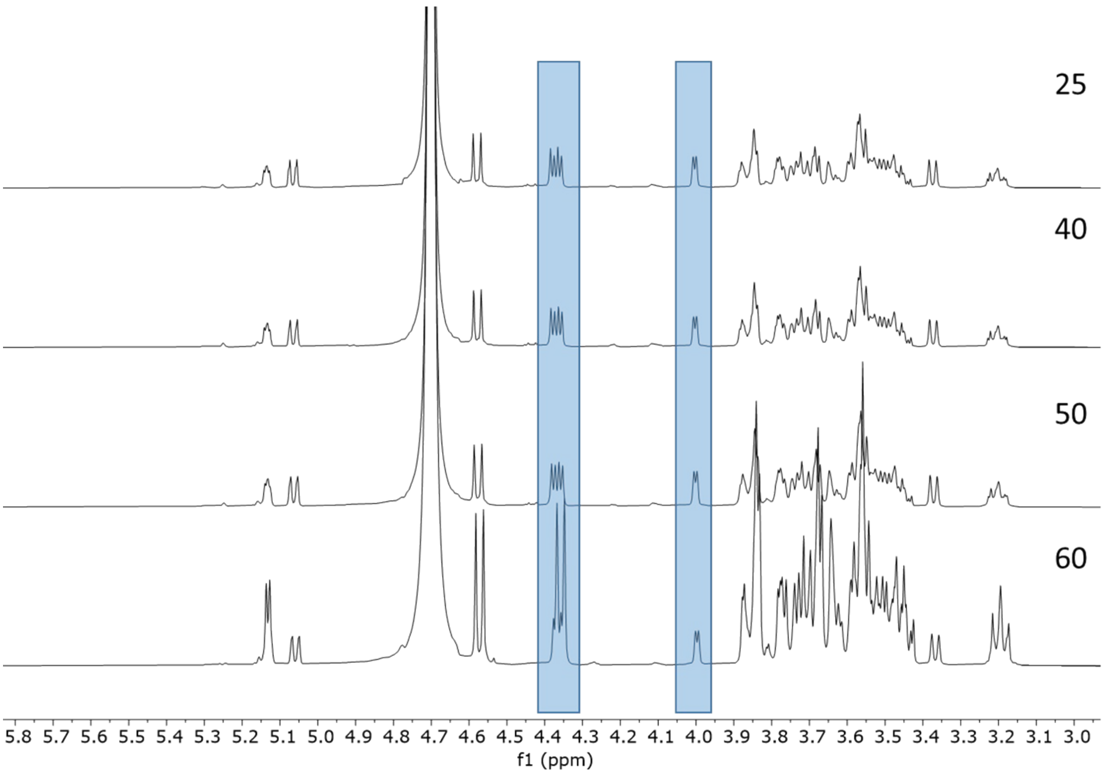

Temp	C1 integ	C4 integ	Conversion (%)
25	1	0.56	56
40	1	0.52	52
50	1	0.46	46
60	1	0.21	21

Figure S8: Thermal stability of free GOase M_1 (1 mg mL^{-1}) in the bio-oxidation of 100 mM lactose.

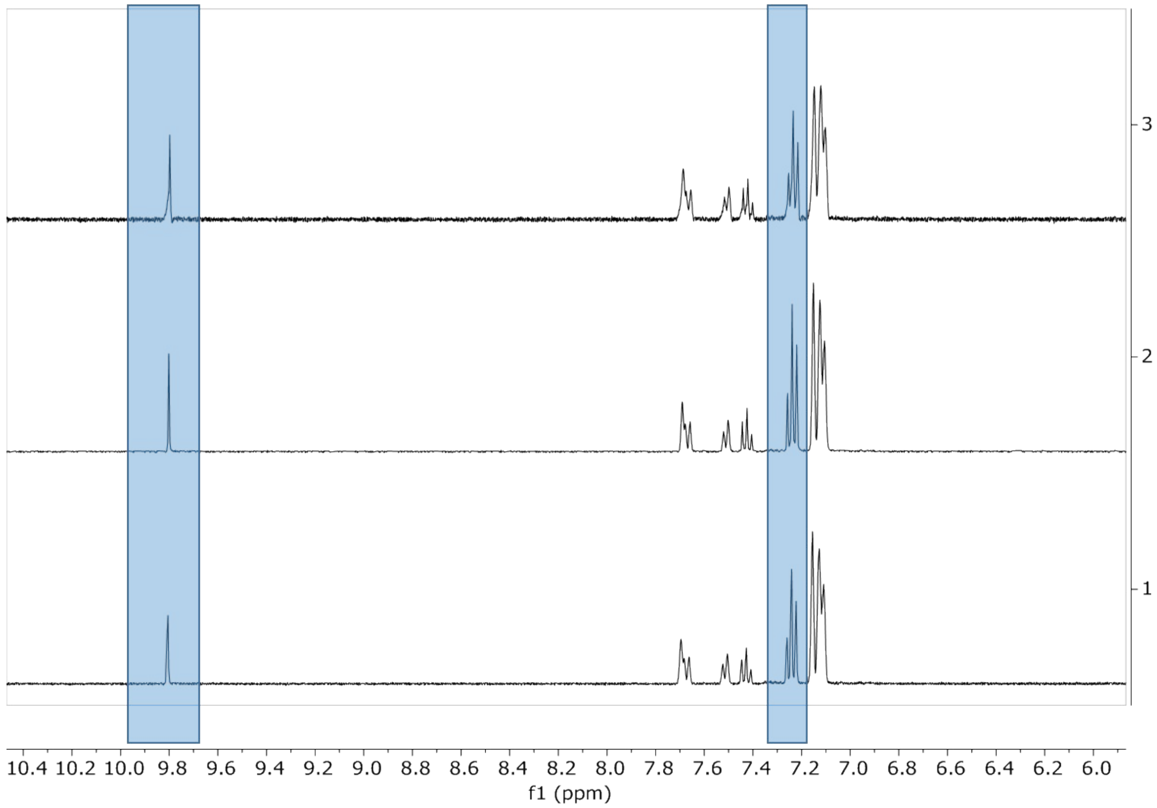

Day	Aldehyde Integ	7.2ppm integ	Conversion (%)
1	1	3.60	21.7
4	1	3.80	20.8
6	1	3.06	24.6

Figure S9: Stability of GOase $M_{3\text{-}5}$ (20 mg, 10 wt%) in the bio-oxidation of 50 mM 3-Me-benzyl alcohol.

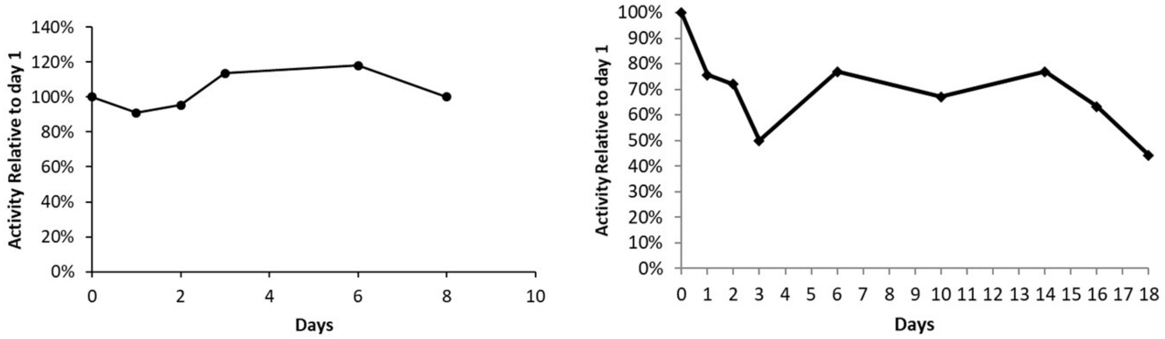

Figure S10: Reuse of immobilised GOase $M_{3\text{-}5}$ (left) and immobilised AcO6 (right) with the bio-oxidation of 50 mM 3-Me-benzyl alcohol (GOase $M_{3\text{-}5}$) and 20 mM 1-hexanol (AcCO6).

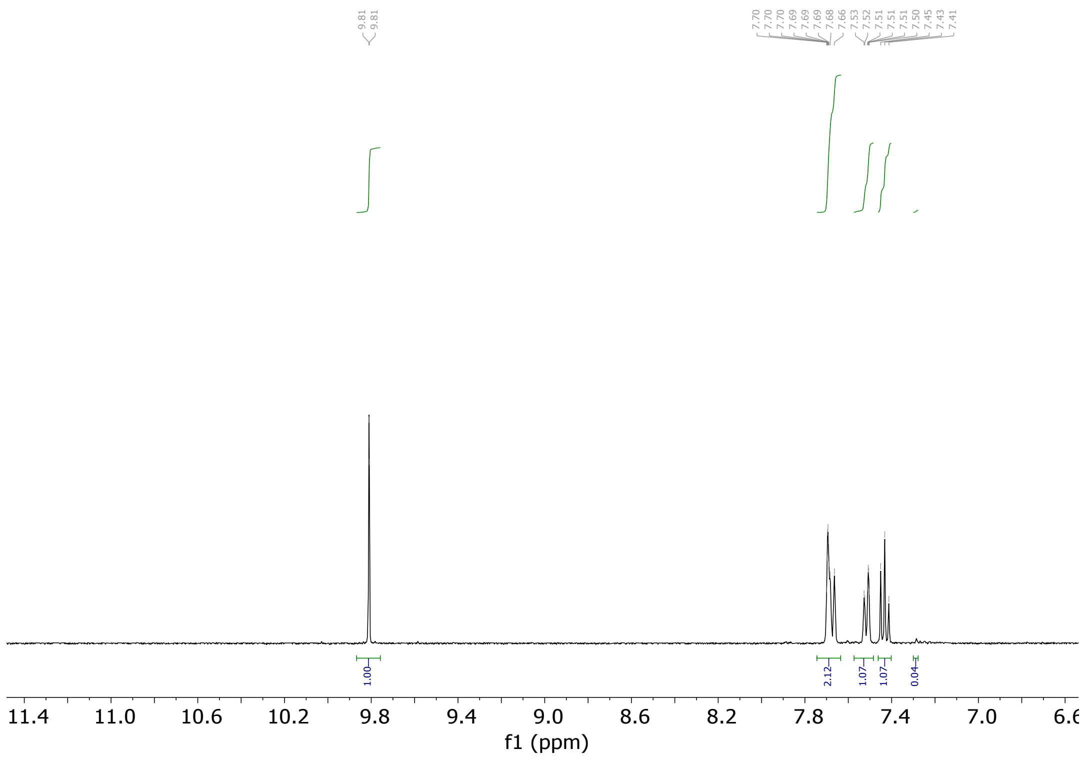

Figure S11: ^{1}H NMR analysis of the bio-oxidation of 25 mM 3-Me-benzyl alcohol with reused immobilised GOase M_{3-5}.

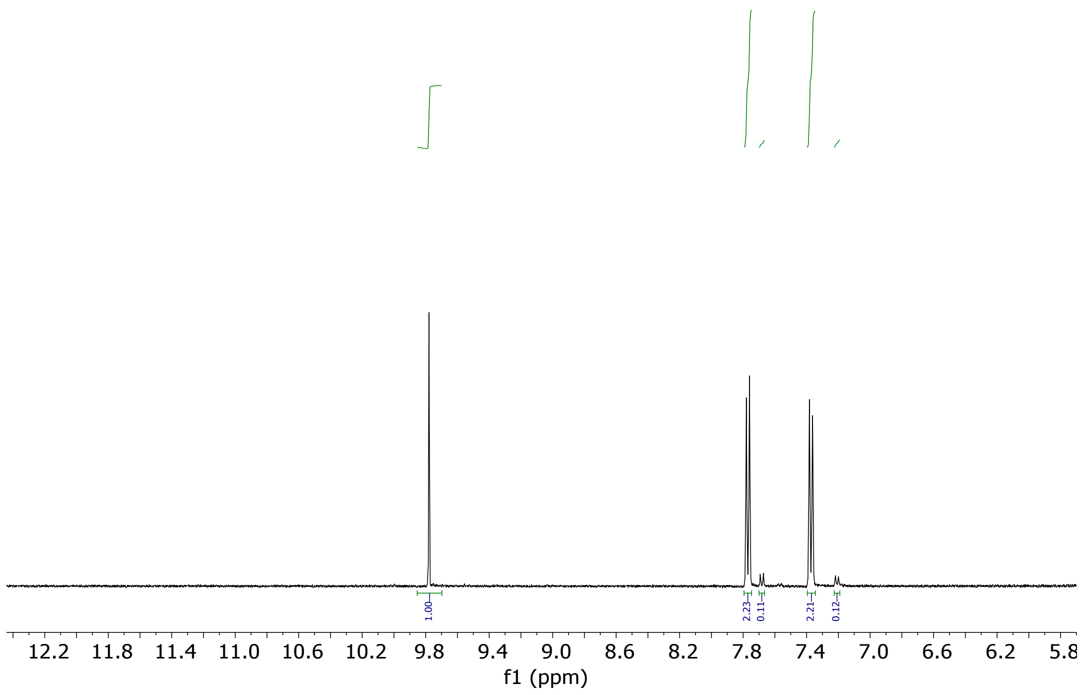

Figure S12: ^{1}H NMR analysis of the bio-oxidation of 25 mM 3-Me-benzyl alcohol with reused immobilised GOase M_{3-5}.

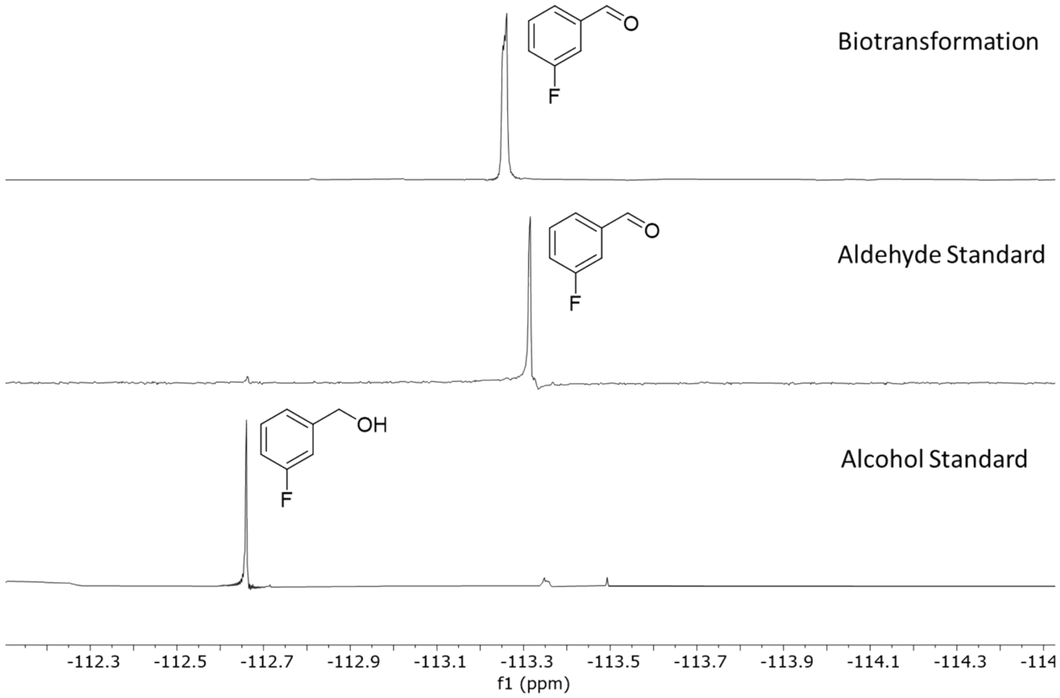

Figure S13: ^{19}F NMR analysis of the bio-oxidation of 25 mM 3-F-benzyl alcohol with reused immobilised GOase M_{3-5}

Substrate	Retention time (min)	Product	Retention time (min)
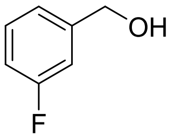	6.57	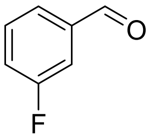	5.91
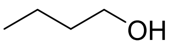	5.11	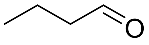	4.62
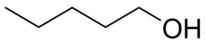	4.65	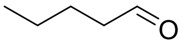	4.44
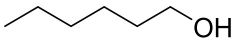	5.26	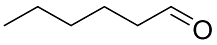	4.63
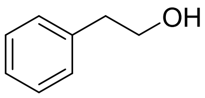	6.30	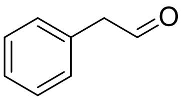	4.68
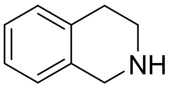	9.68	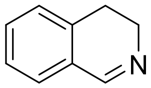	9.55
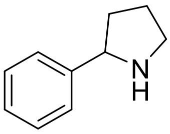	9.86	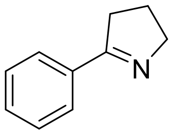	10.06
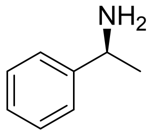	11.94	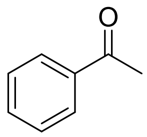	12.09
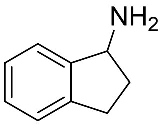	7.46	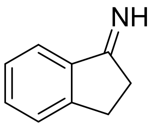	7.70

Table S1: GC-FID Retention times for oxidase substrates and products

Solvent	Conversion (%)			Relative Conversion to buffer		
	GOase	Chol Ox	MAO	Goase	Chol Ox	MAO
Buffer	70	40.882	48	1	1	1
EtOAc	7.2	24.002	11	0.10286	0.5871043	0.229167
Hexane	61.5	18.422	14	0.87857	0.450614	0.291667
Cyclohexane	42.6	28	13	0.60857	0.2708333	0.270833
Toluene	59.6	13.019	12	0.85143	0.3184531	0.25
MeTHF	2.8	6.286	12	0.04	0.1537596	0.25

Table S2: Conversions with immobilised oxidases in neat organic solvent. This table is a numerical representation of figure 4 in the manuscript.

GC traces

GOase M_{3-5} Solvent screen

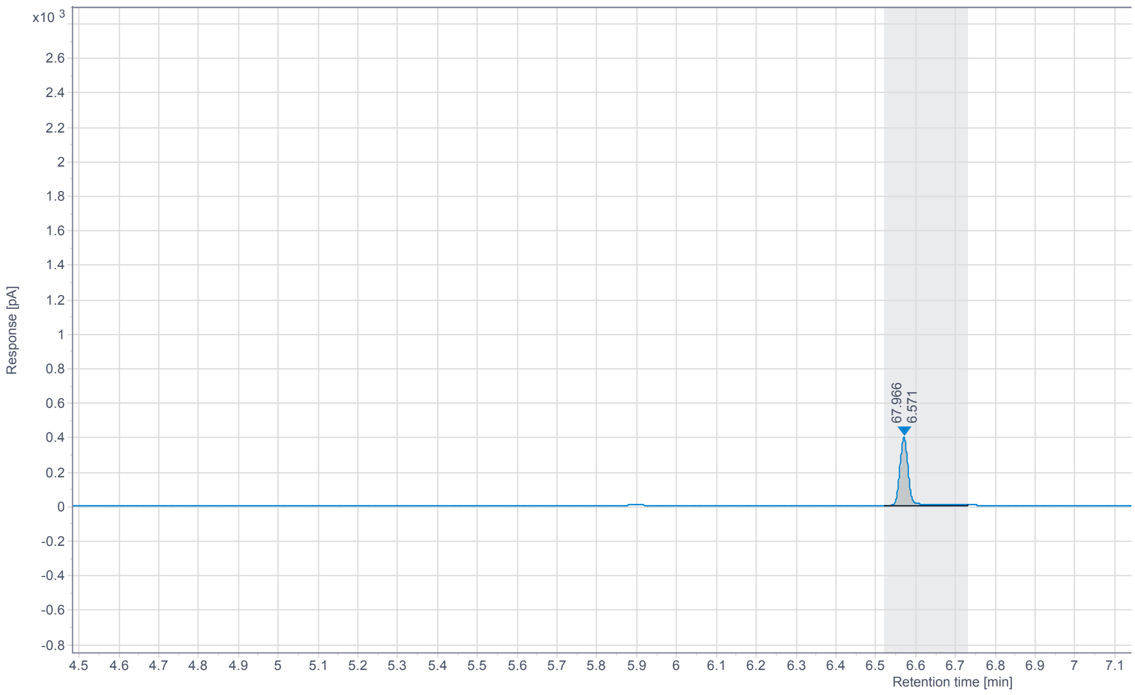

Figure S14: Analytical standard of 3-F-benzyl alcohol.

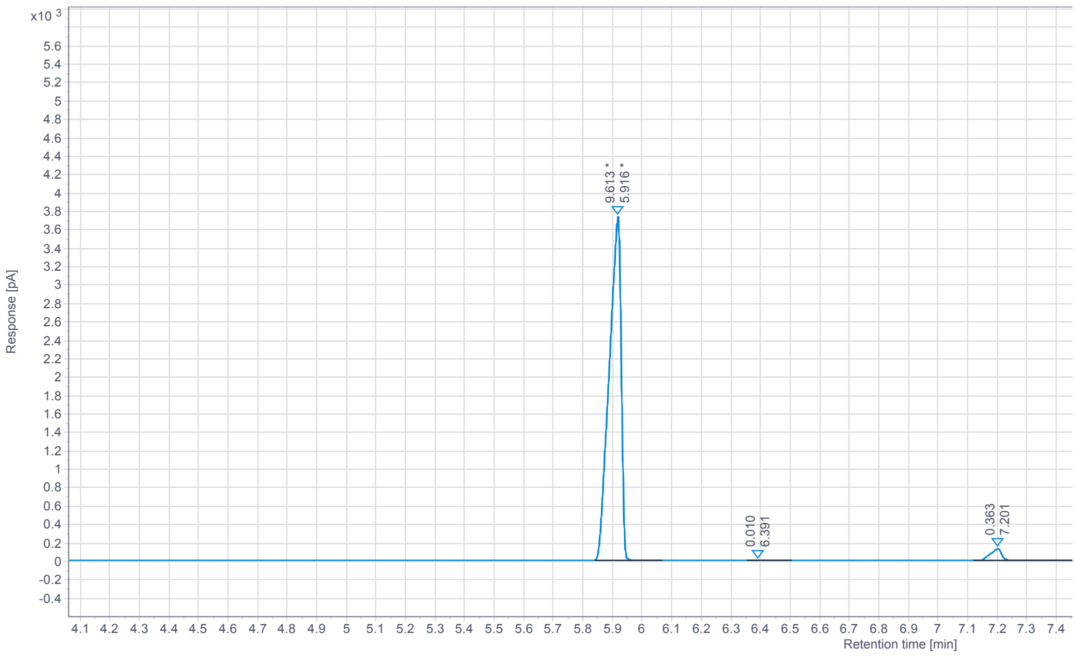

Figure S15: Analytical standard of 3-F-benzaldehyde.

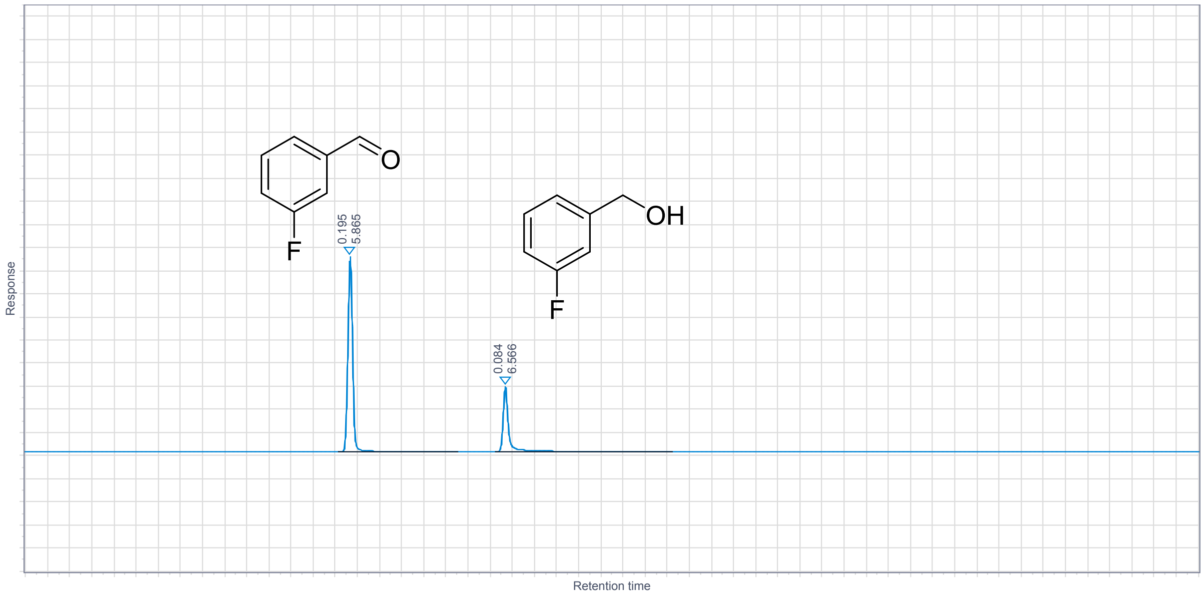

Figure S16: Immobilised GOase $M_{3\text{-}5}$ bio-oxidation of 25 mM 3-F-benzyl alcohol in NaPi buffer (100 mM, pH 7.4).

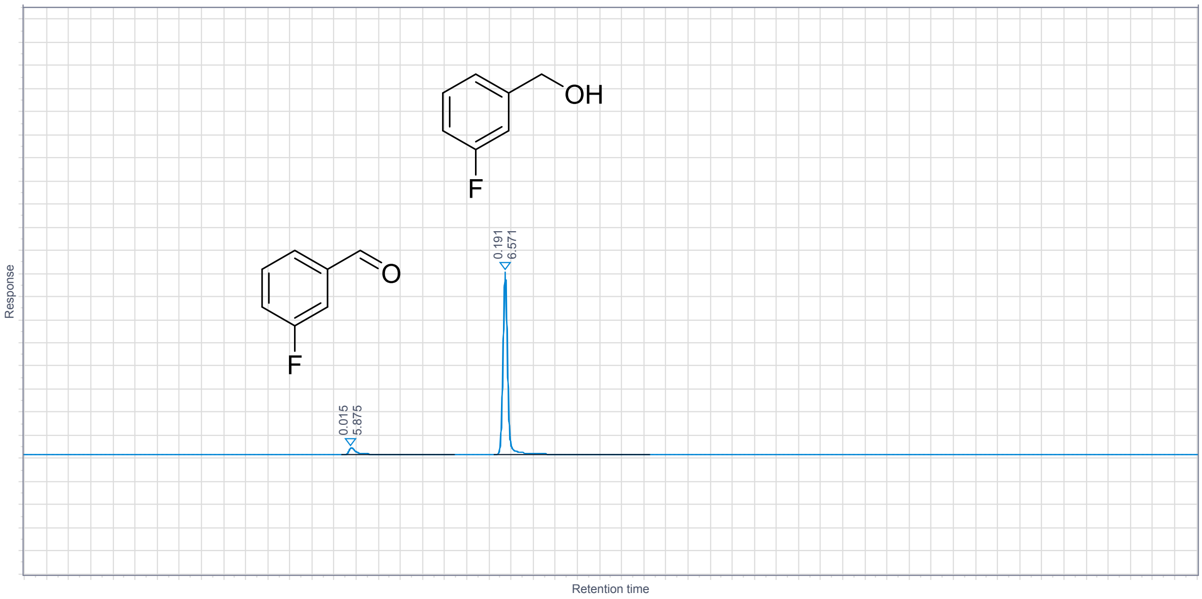

Figure S17: Immobilised GOase $M_{3\text{-}5}$ bio-oxidation of 25 mM 3-F-benzyl alcohol in EtOAc.

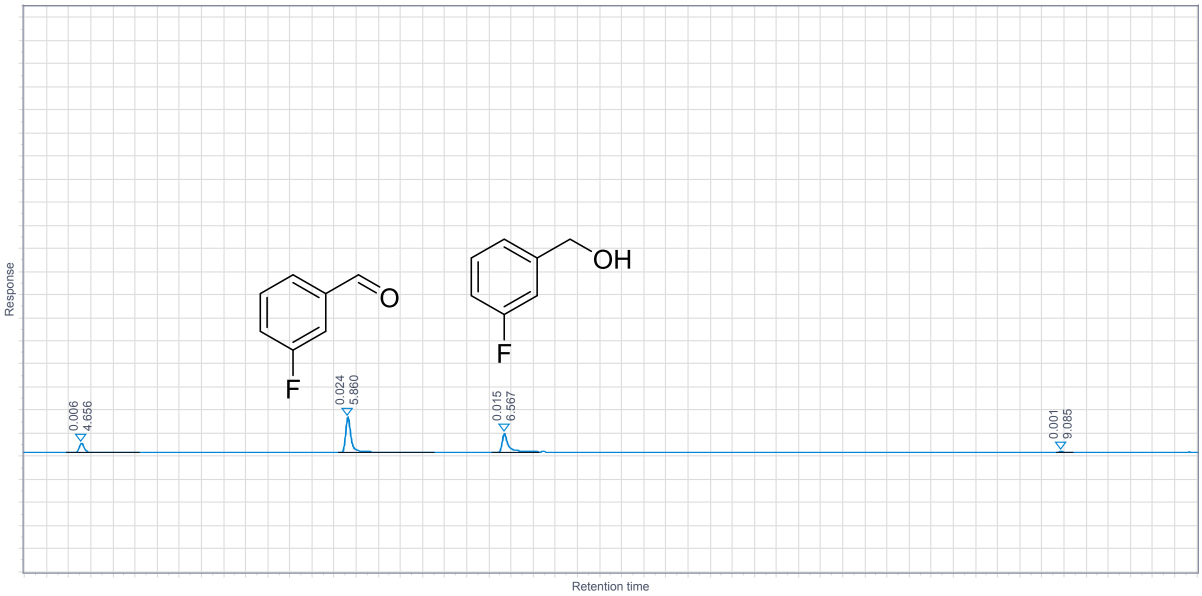

Figure S18: Immobilised GOase $M_{3\text{-}5}$ bio-oxidation of 25 mM 3-F-benzyl alcohol in hexane.

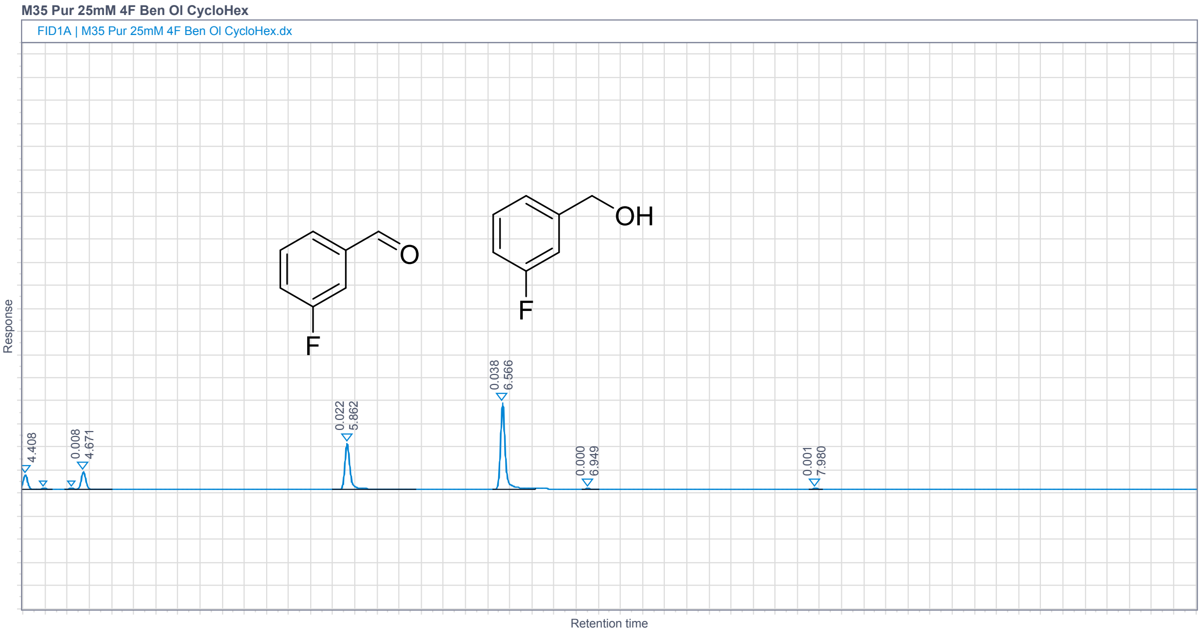

Figure S19: Immobilised GOase M_{3-5} bio-oxidation of 25 mM 3-F-benzyl alcohol in cyclohexane

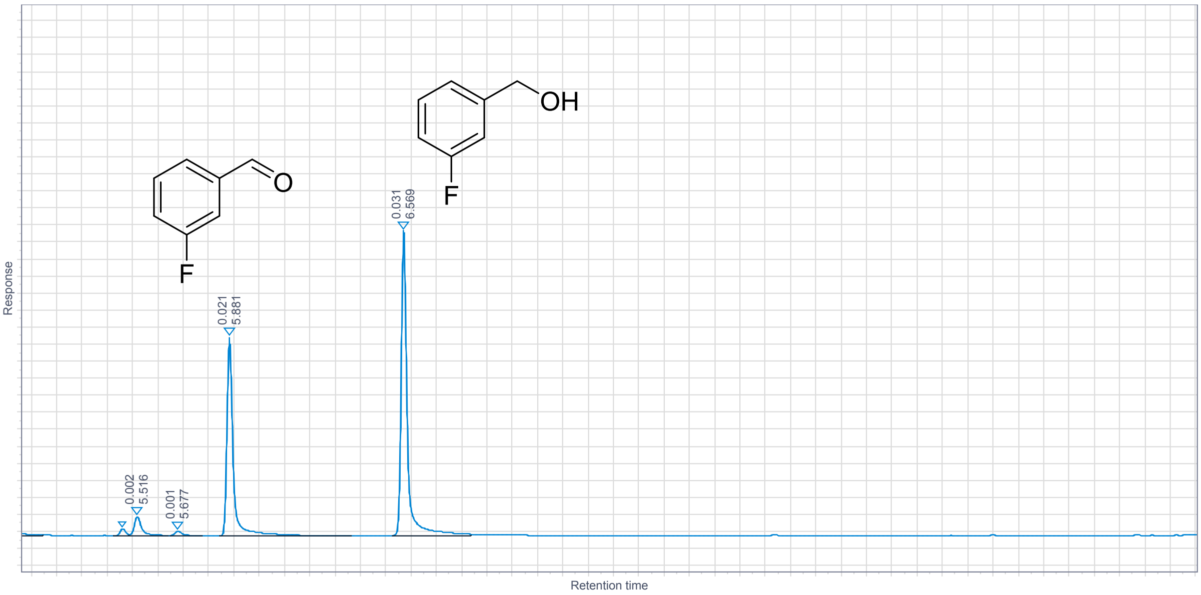

Figure S20: Immobilised GOase M_{3-5} bio-oxidation of 25 mM 3-F-benzyl alcohol in toluene

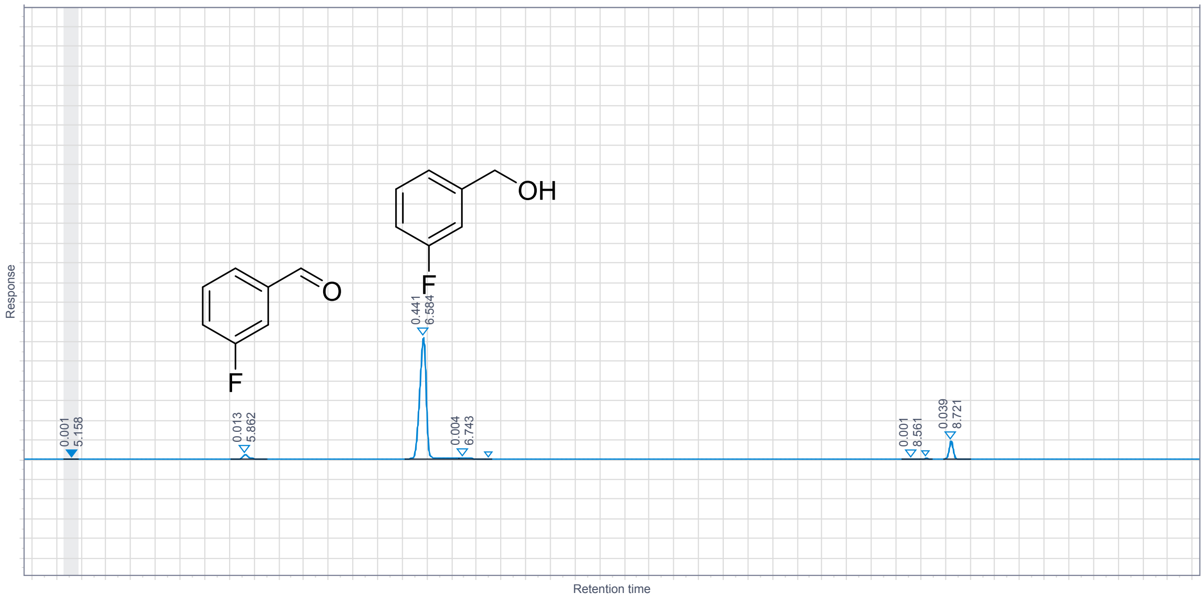

Figure S21: Immobilised GOase M_{3-5} bio-oxidation of 25 mM 3-F-benzyl alcohol in 2-MeTHF

Monoamine Oxidase solvent screen

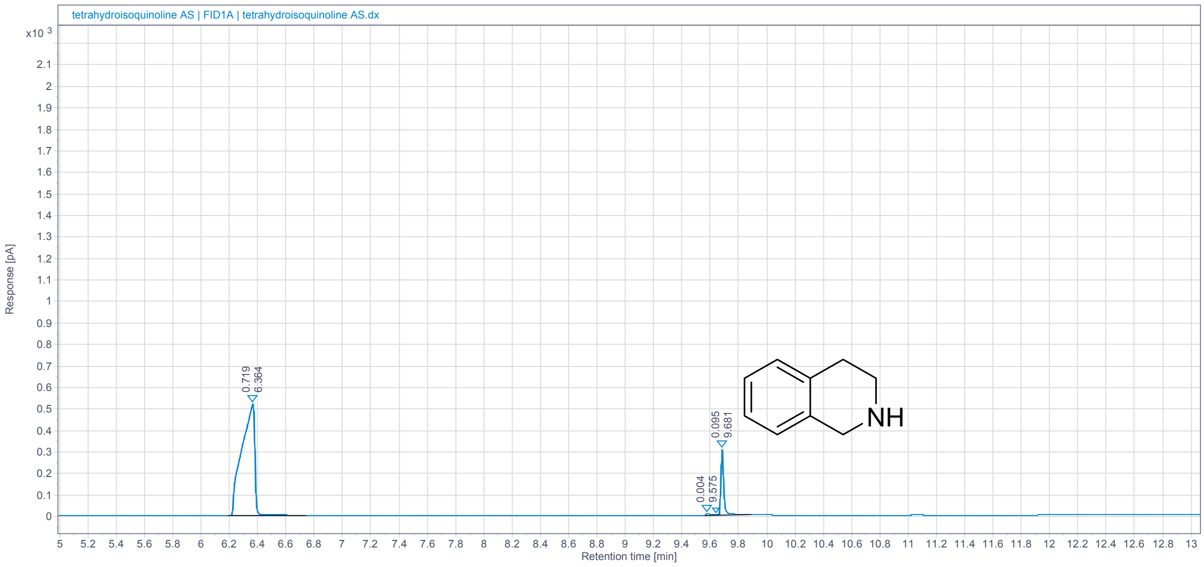

Figure S22: Analytical standard of 1,2,3,4-tetrahydroisoquinoline (THIQ).

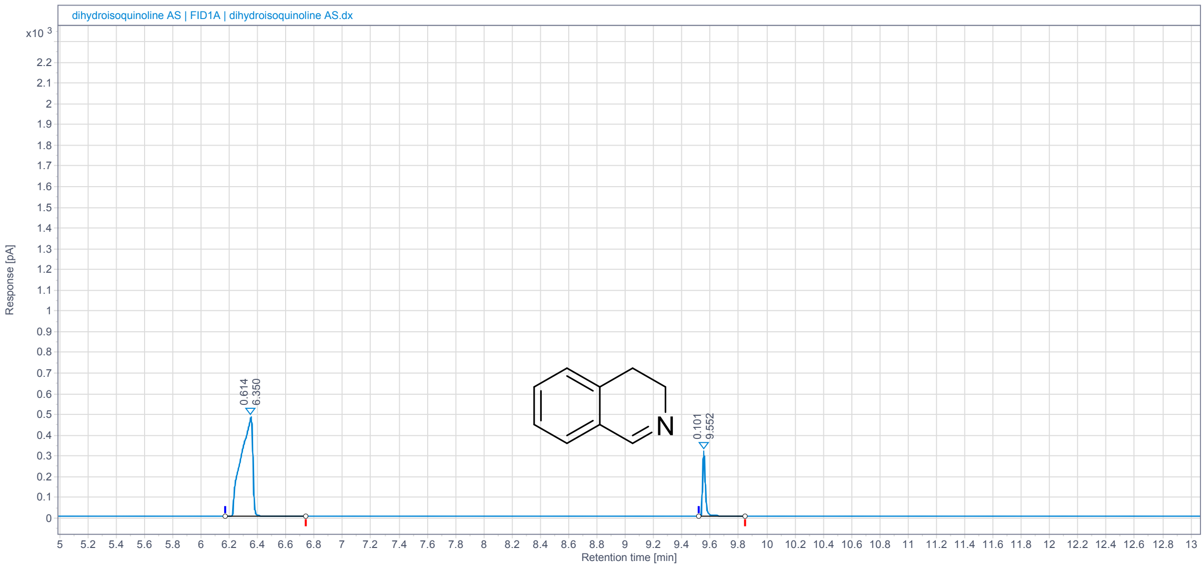

Figure S23: Analytical standard of 3,4-dihydroisoquinoline (DHIQ).

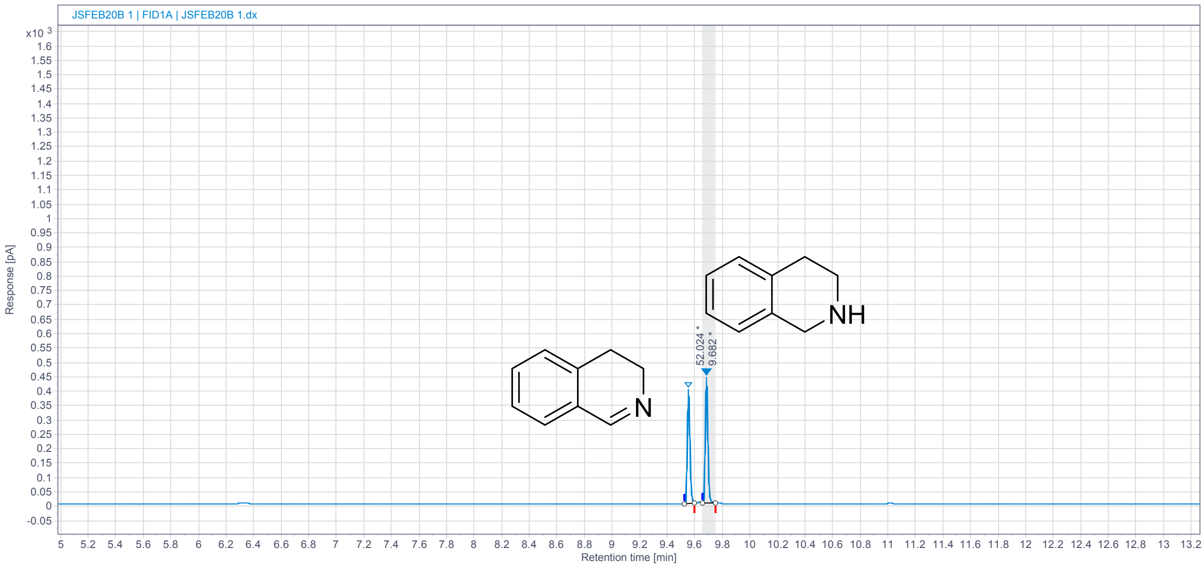

Figure S24: Immobilised MAO-N D9 bio-oxidation of 20mM THIQ in KPi buffer (100 mM, pH 8).

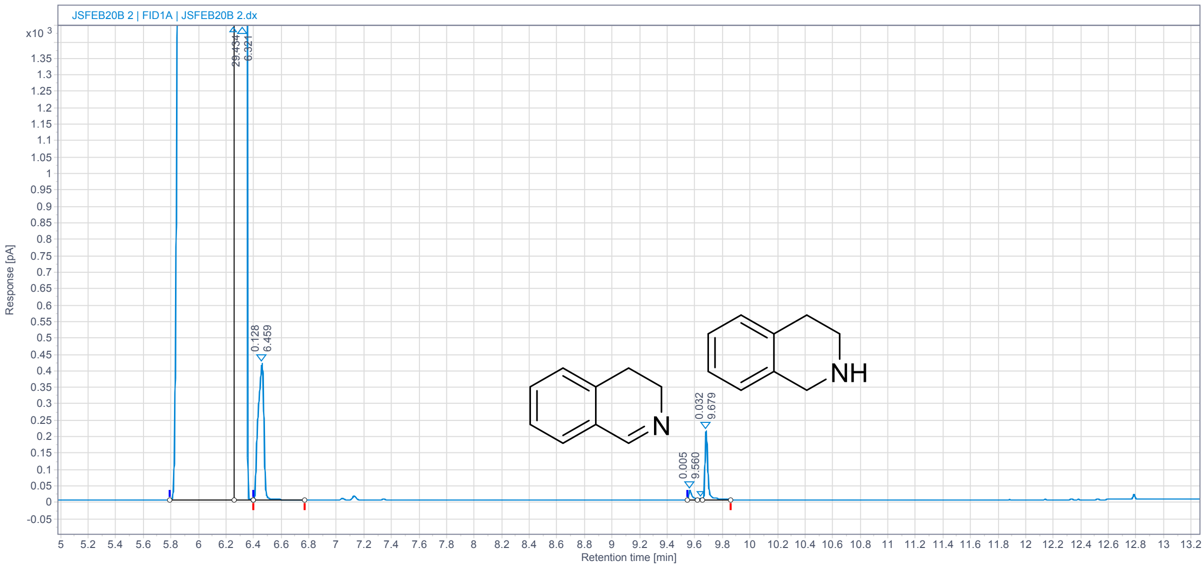

Figure S25: Immobilised MAO-N D9 bio-oxidation of 20 mM THIQ in toluene.

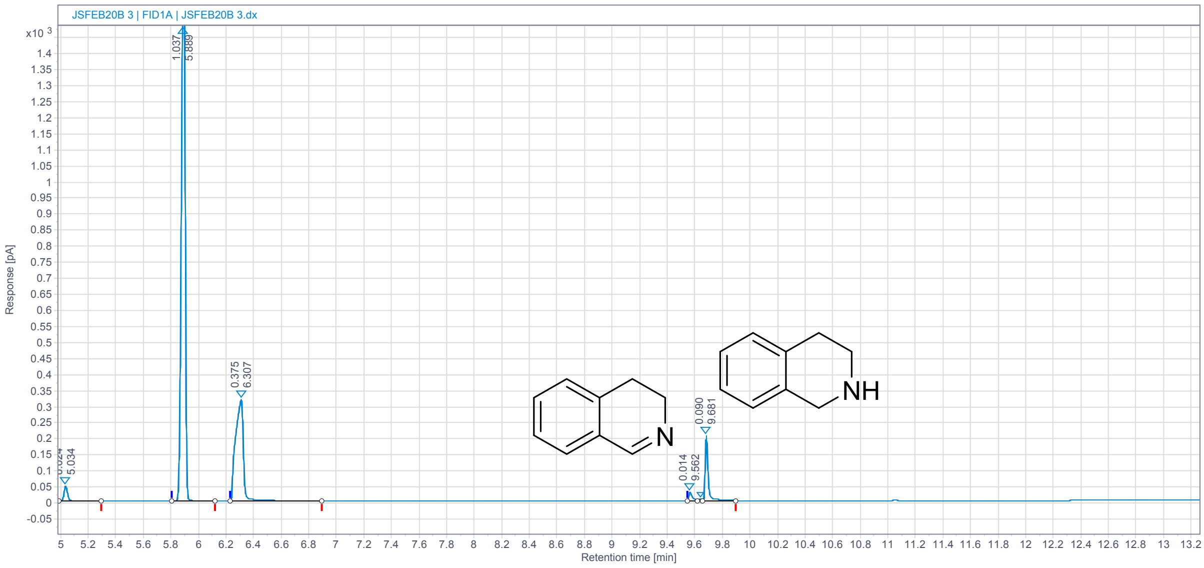

Figure S26: Immobilised MAO-N D9 bio-oxidation of 20 mM THIQ in EtOAc.

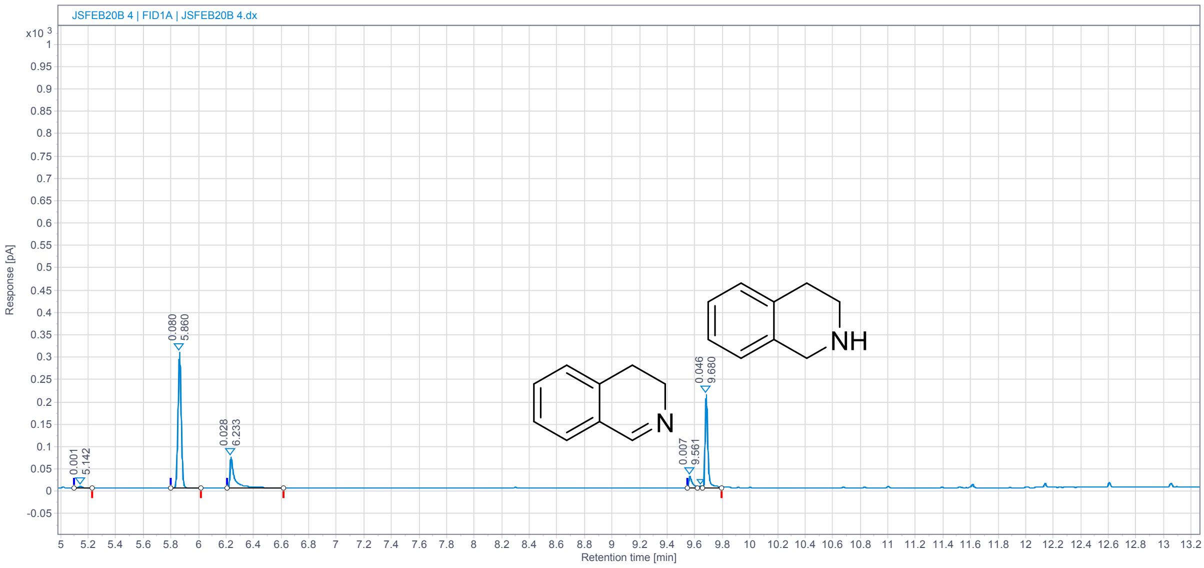

Figure S27: Immobilised MAO-N D9 bio-oxidation of 20 mM THIQ in hexane.

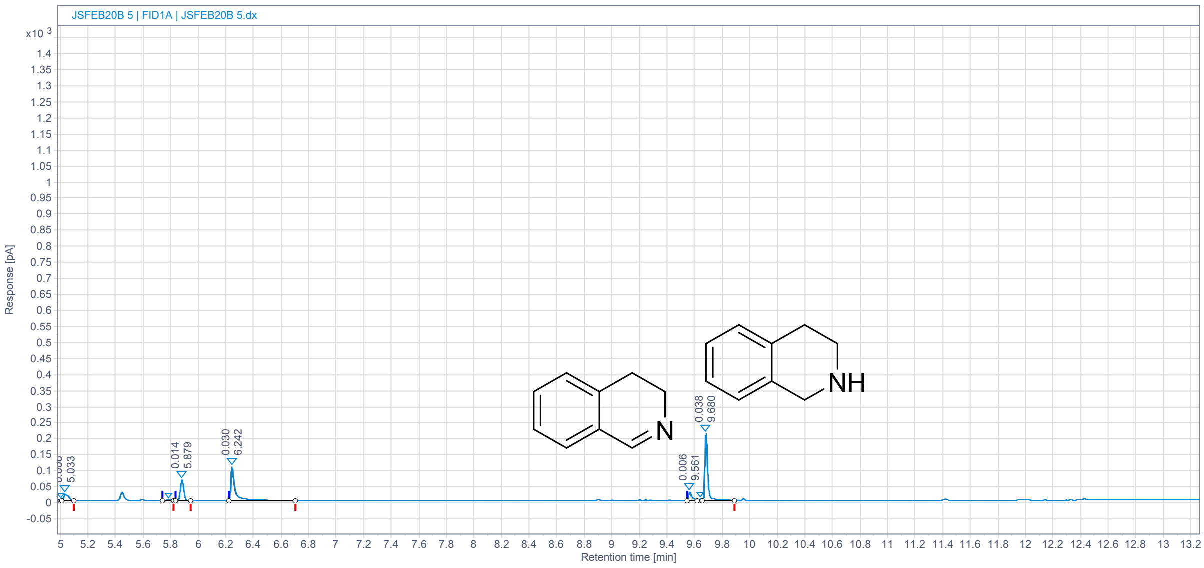

Figure S28: Immobilised MAO-N D9 bio-oxidation of 20 mM THIQ in cyclohexane.

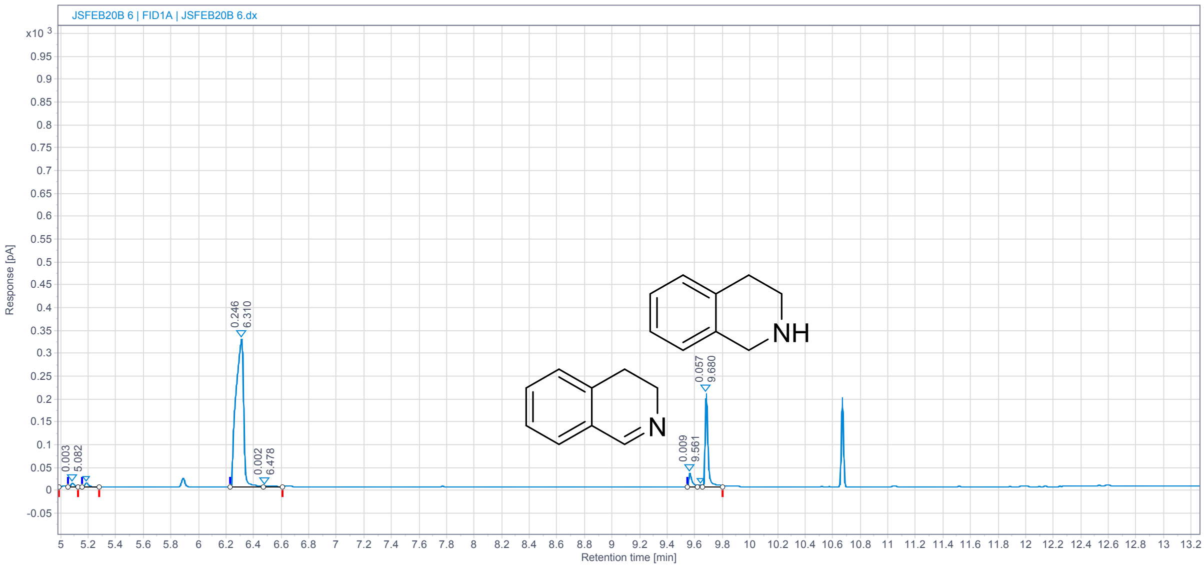

Figure S29: Immobilised MAO-N D9 bio-oxidation of 20 mM THIQ in 2-MeTHF.

Choline Oxidase heterogeneous Biotransformations

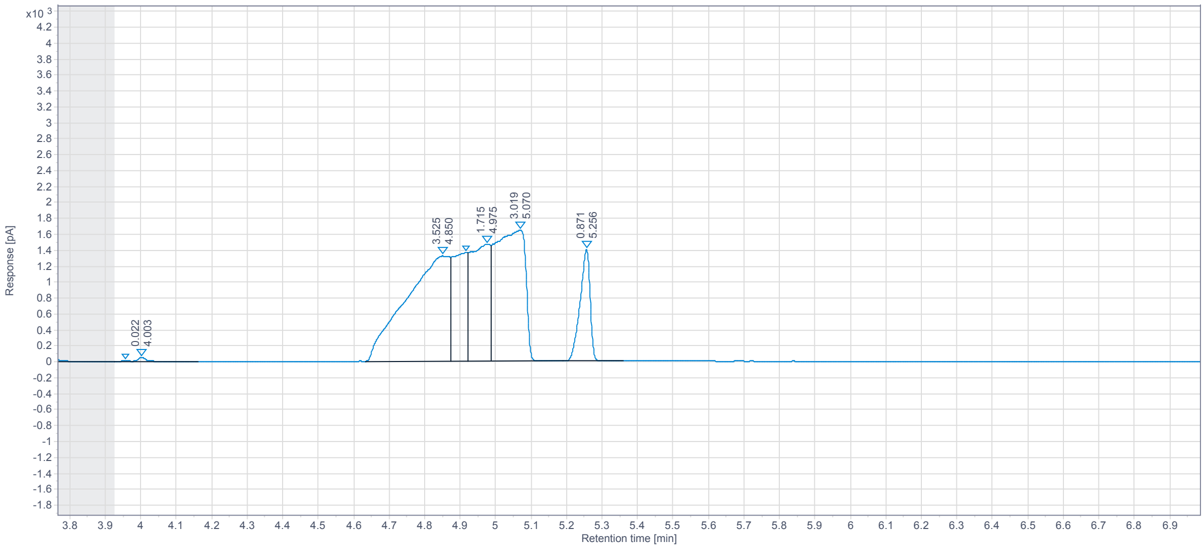

Figure S30: Analytical standard of 1-hexanol.

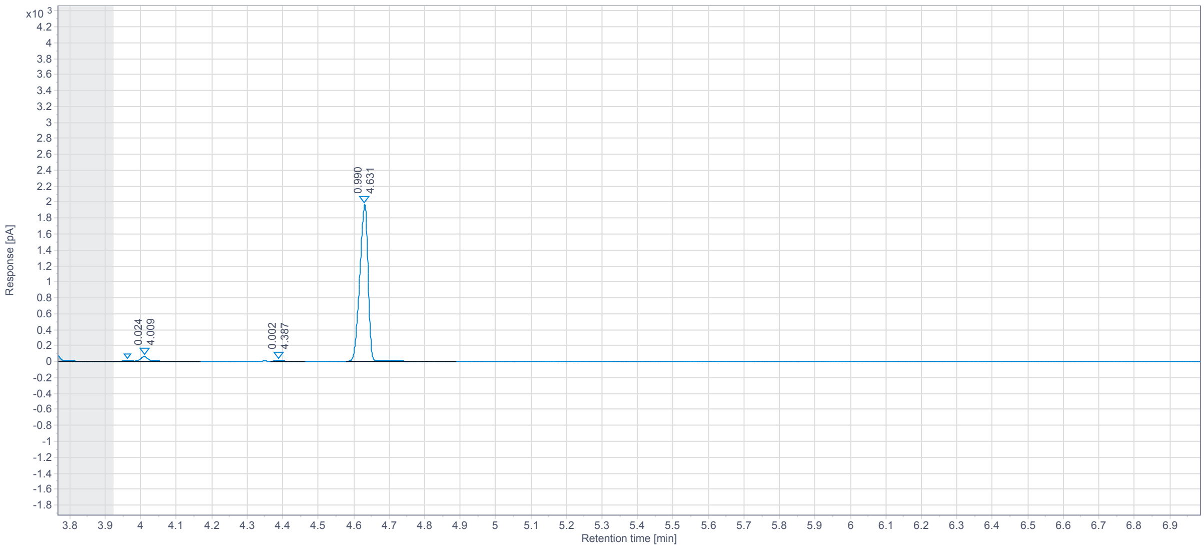

Figure S31: Analytical standard of 1-hexanal.

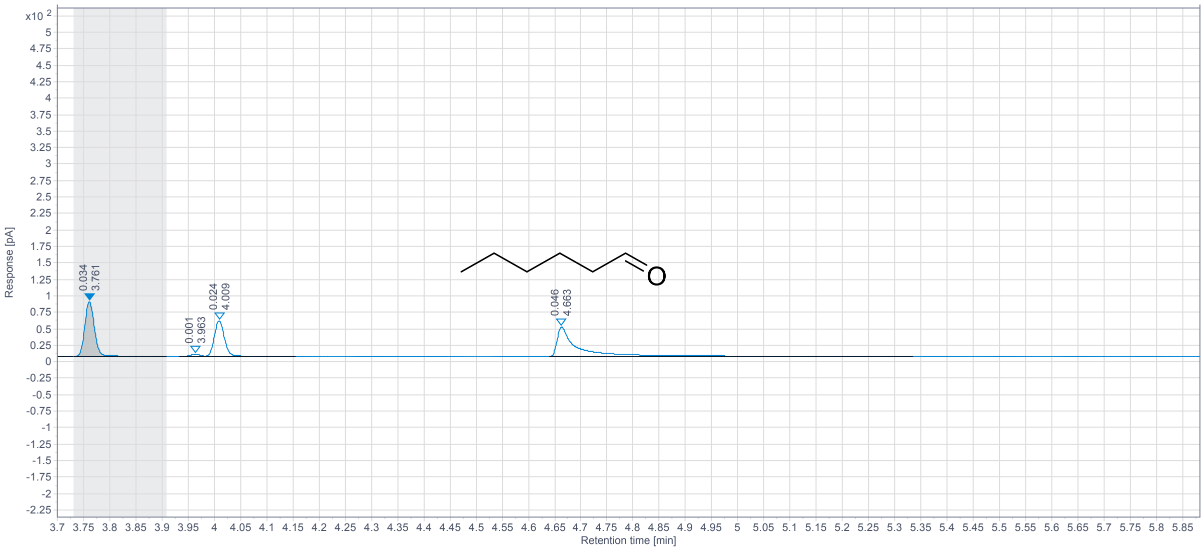

Figure S32: Immobilised AcCO6 4 h bio-oxidation of 10 mM 1-hexanol in KPi buffer (100 mM, pH 8).

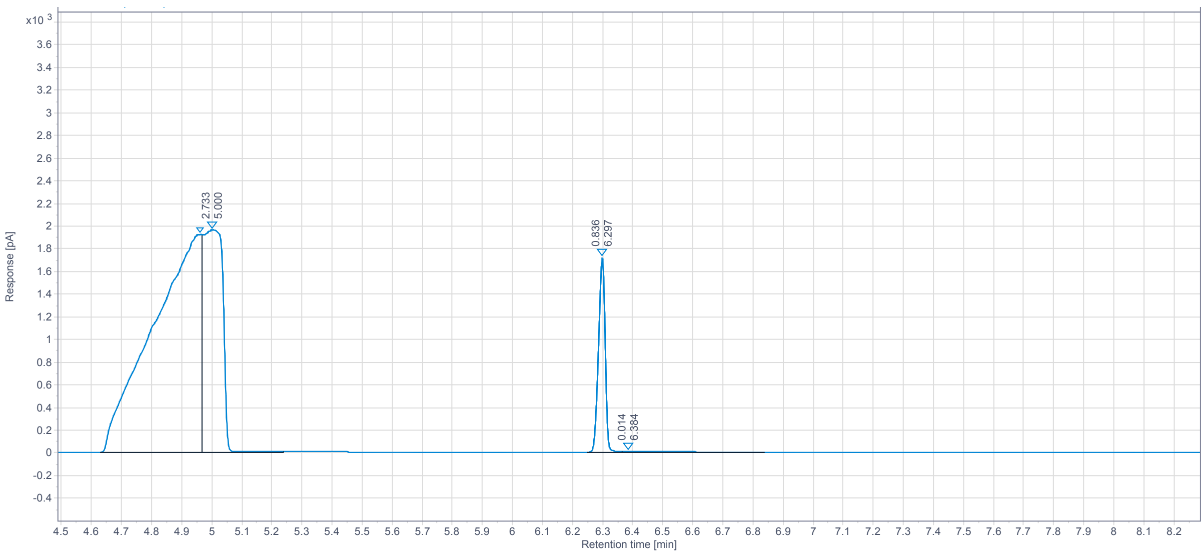

Figure S33: Analytical standard of 2-phenylethanol.

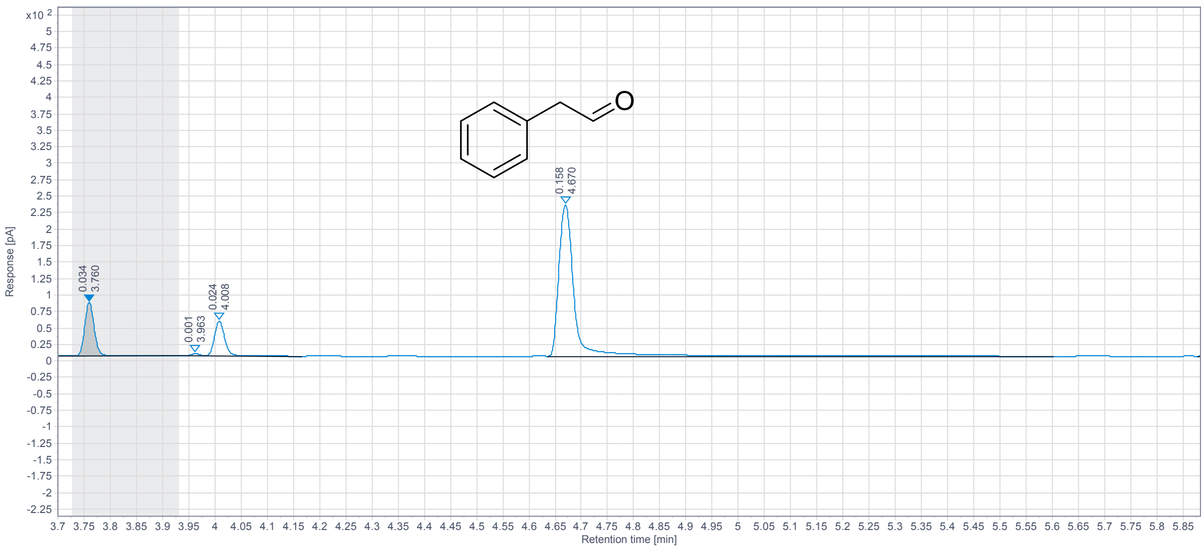

Figure S34: Immobilised AcCO6 4 h bio-oxidation of 10 mM 2-phenylethanol in KPi buffer (100 mM, pH 8).

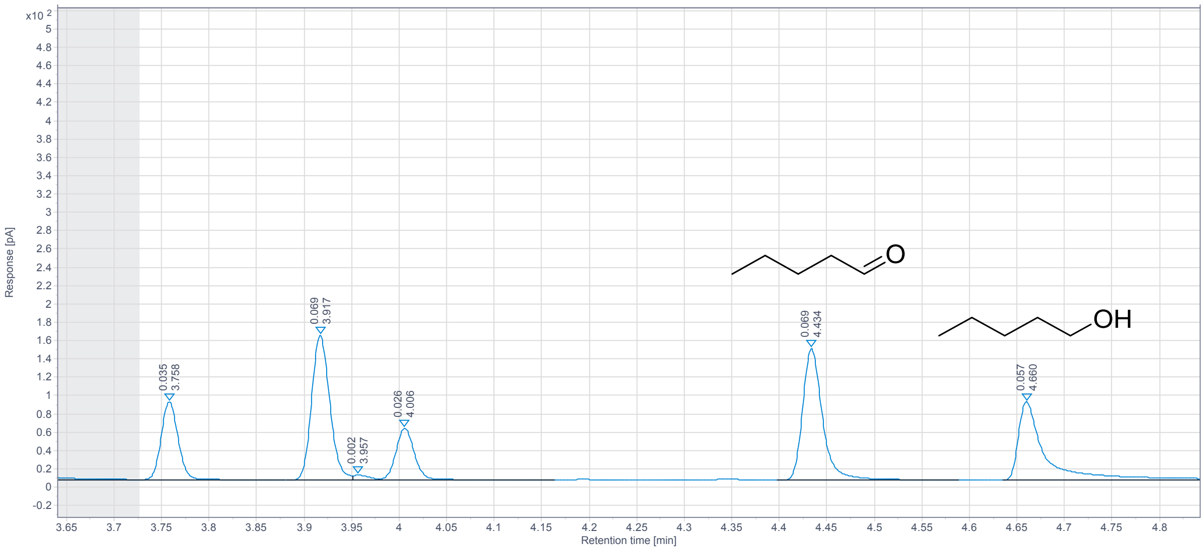

Figure S35: Immobilised AcCO6 4 h bio-oxidation of 10 mM 1-pentanol in KPi buffer (100 mM, pH 8).

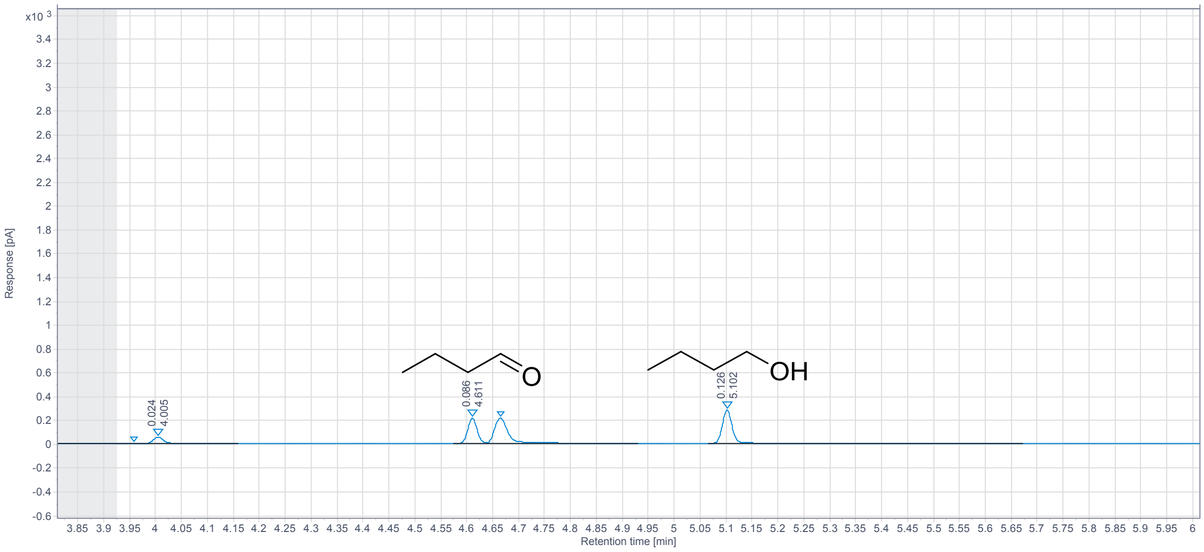

Figure S36: Immobilised AcCO6 4 h bio-oxidation of 10 mM 1-butanol in KPi buffer (100 mM, pH 8).

Monoamine Oxidase heterogeneous biotransformations

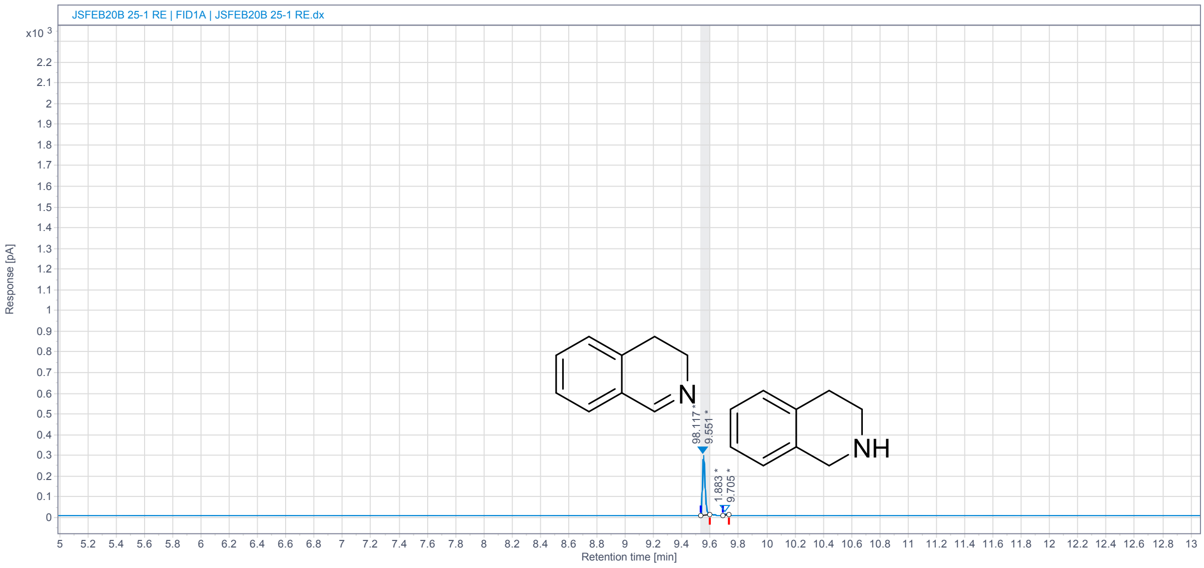

Figure S37: Immobilised MAO-N D9 4 h bio-oxidation of 20 mM THIQ in KPi buffer (100 mM, pH 7.8).

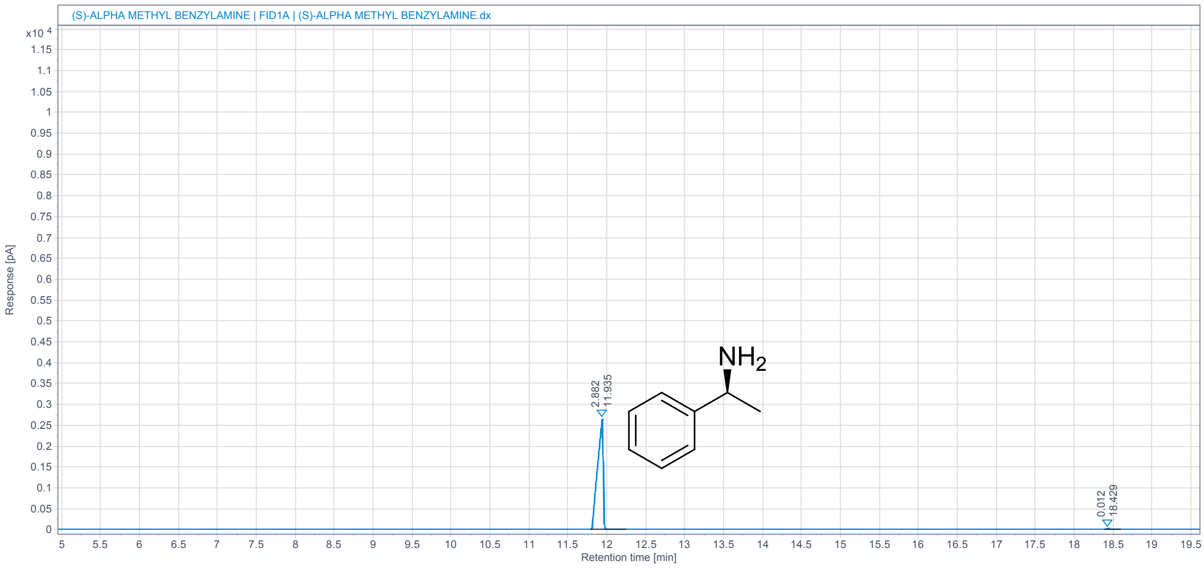

Figure S38: Analytical standard of (*S*)-α-methyl benzylamine.

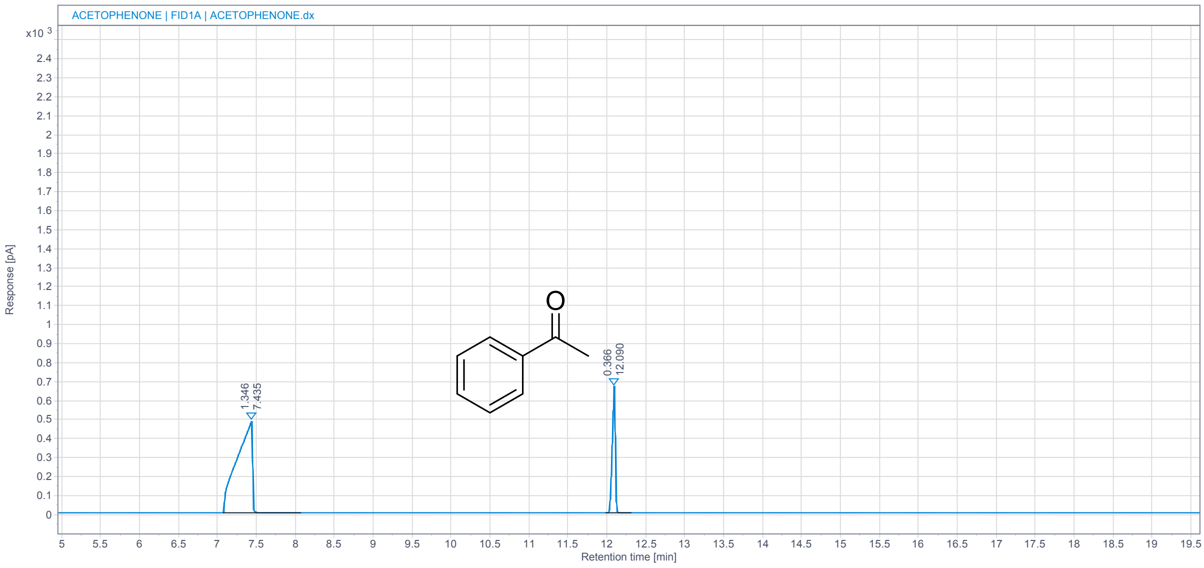

Figure S39: Analytical standard of acetophenone.

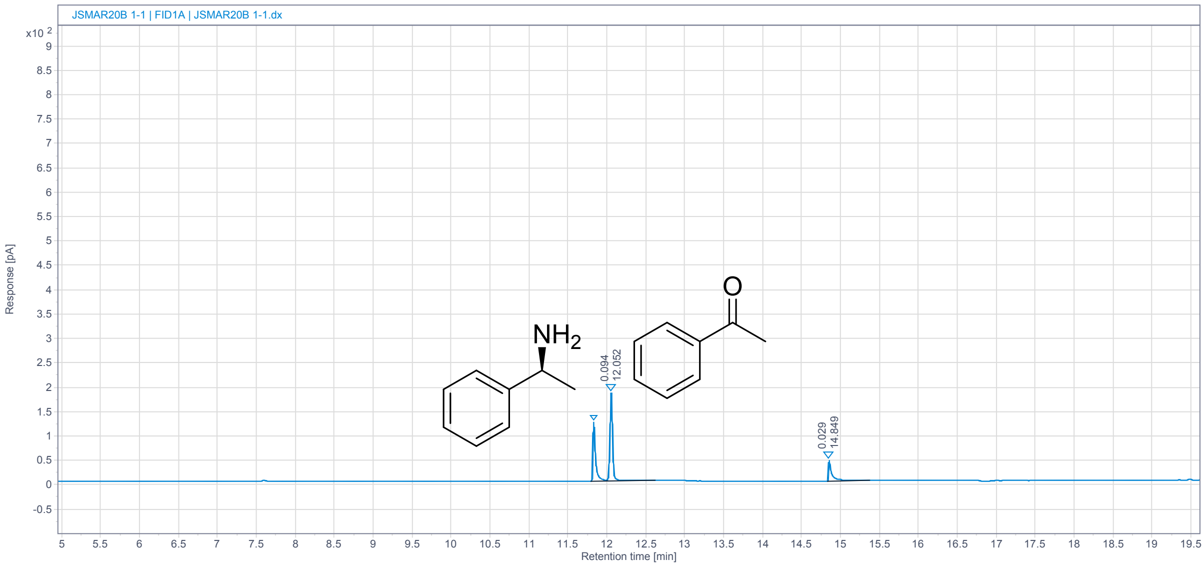

Figure S40: Immobilised MAO-N D9 4 h bio-oxidation of 20 mM (*S*)-alpha-methyl benzylamine in KPi buffer (100 mM, pH 7.8).

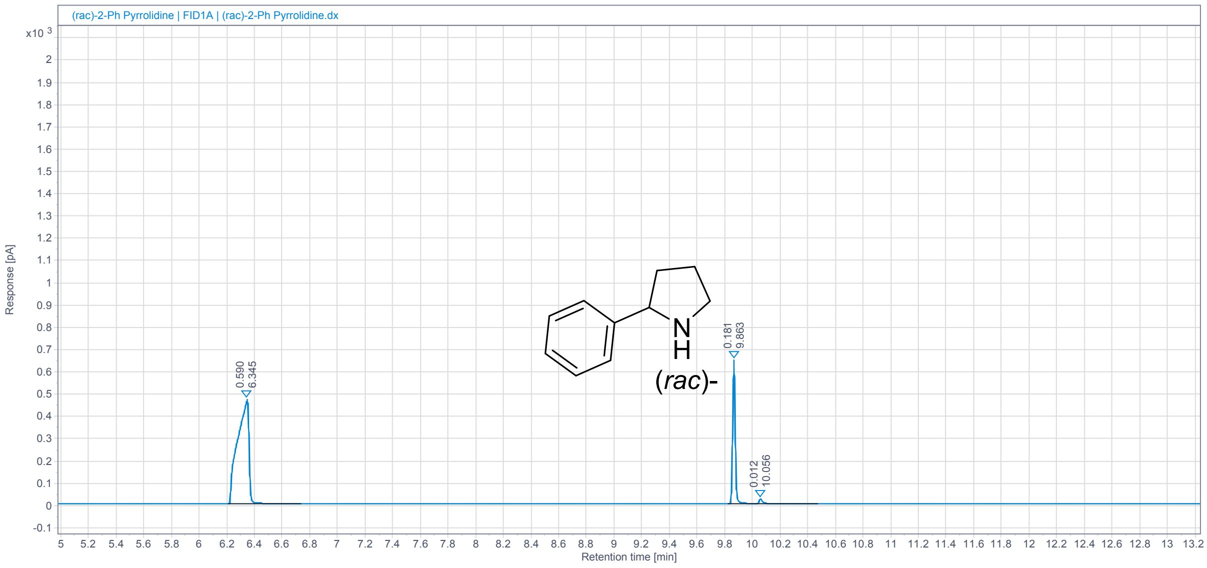

Figure S41: Analytical standard of (*rac*)-2-phenylpyrrolidine.

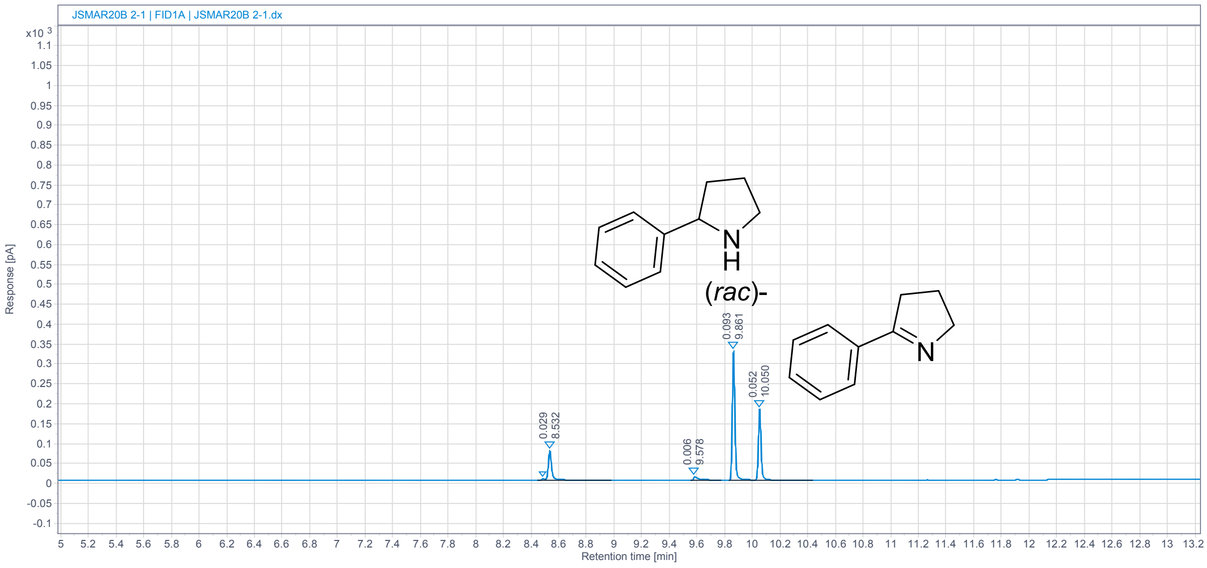

Figure S42: Immobilised MAO-N D9 4 h bio-oxidation of 20 mM (*rac*)-2-phenylpyrrolidine in KPi buffer (100 mM, pH 7.8).

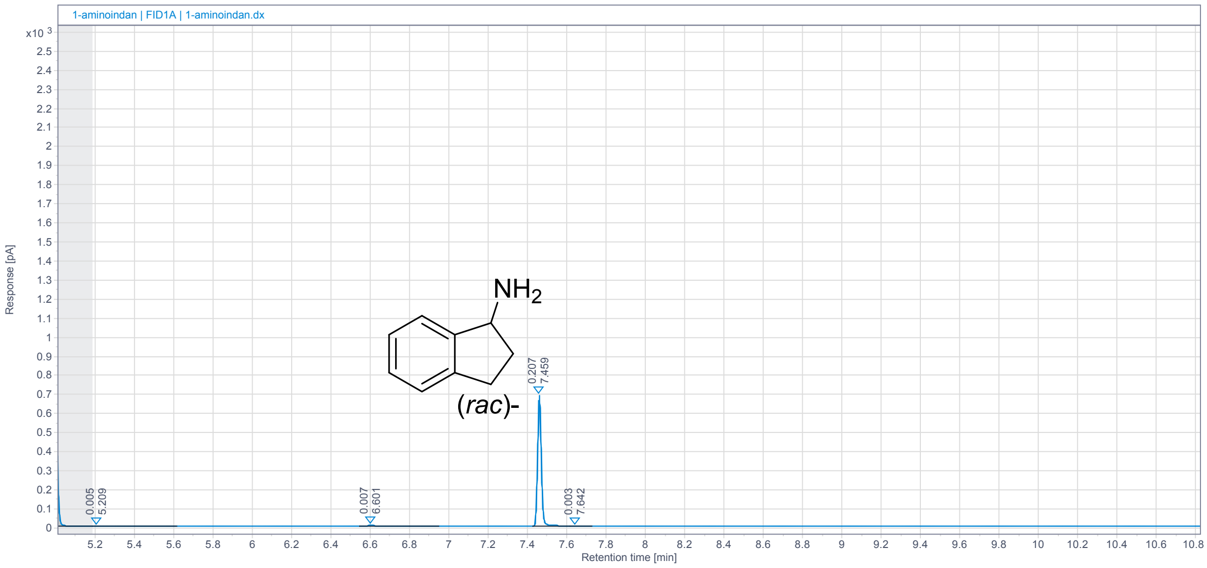

Figure S43: Analytical standard of (*rac*)-1-aminoindane.

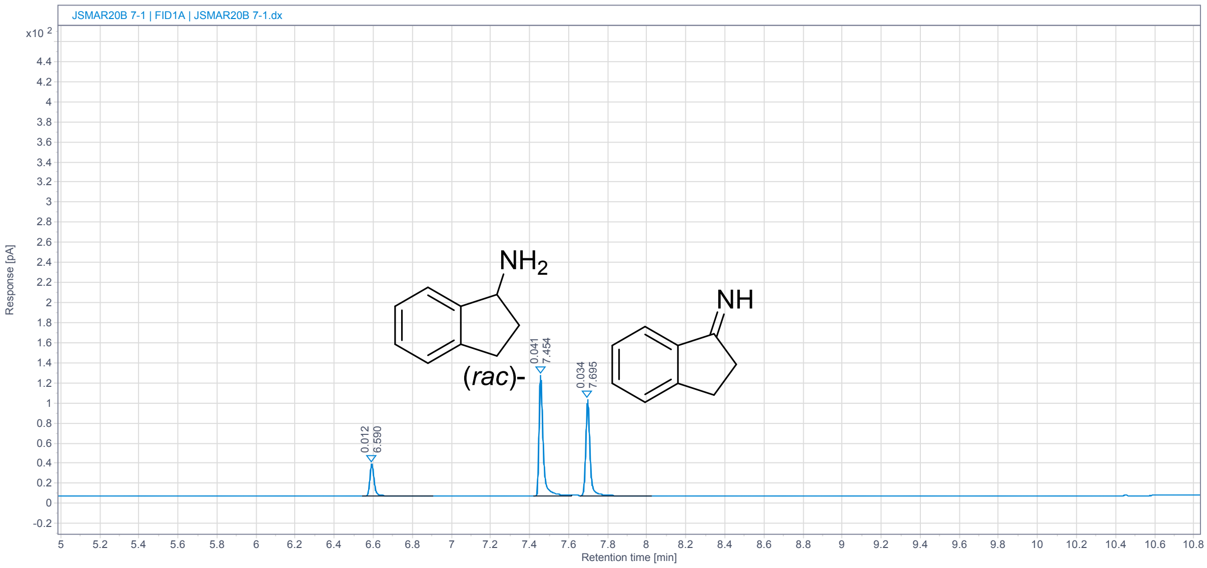

Figure S44: Immobilised MAO-N D9 4 h bio-oxidation of 20 mM (*rac*)-1-aminoindane in KPi buffer (100 mM, pH 7.8).

References

(1) Toftgaard Pedersen, A.; Birmingham, W. R.; Rehn, G.; Charnock, S. J.; Turner, N. J.; Woodley, J. M. Process Requirements of Galactose Oxidase Catalyzed Oxidation of Alcohols. *Org. Process Res. Dev.* **2015**, *19* (11), 1580–1589. https://doi.org/10.1021/acs.oprd.5b00278.

(2) Heath, R. S.; Birmingham, W. R.; Thompson, M. P.; Taglieber, A.; Daviet, L.; Turner, N. J. An Engineered Alcohol Oxidase for the Oxidation of Primary Alcohols. *ChemBioChem* **2019**. https://doi.org/10.1002/cbic.201800556.

(3) Cosgrove, S. C.; Hussain, S.; Turner, N. J.; Marsden, S. P. Synergistic Chemo/Biocatalytic Synthesis of Alkaloidal Tetrahydroquinolines. *ACS Catal.* **2018**. https://doi.org/10.1021/acscatal.8b01220.

(4) Rannes, J. B.; Ioannou, A.; Willies, S. C.; Grogan, G.; Behrens, C.; Flitsch, S. L.; Turner, N. J. Glycoprotein Labeling Using Engineered Variants of Galactose Oxidase Obtained by Directed Evolution. *J. Am. Chem. Soc.* **2011**, *133* (22), 8436–8439. https://doi.org/10.1021/ja2018477.
